# Supplementary material for: Mediating trade-off between activity and selectivity in alkynes semi-hydrogenation via a hydrophilic polar layer
Source: Nat Commun. 2024 Feb 9;15:1228. doi: 10.1038/s41467-024-45104-6 (PMC10858237; doi:10.1038/s41467-024-45104-6)
Supplement: Supplementary file 1 — Supplementary Information-20240111.docx [file 41467_2024_45104_MOESM1_ESM.docx]

# Supplementary Information

**Mediating trade-off between activity and selectivity in alkynes semi-hydrogenation via a hydrophilic polar layer**

Jinqi Xiong^1^, Shanjun Mao^1^*, Qian Luo^1^, Honghui Ning^1^, Bing Lu^1^, YanLing Liu^1^ and Yong Wang^1^*

*^1^Advanced Materials and Catalysis Group, Center of Chemistry for Frontier Technologies, State Key Laboratory of Clean Energy Utilization, Institute of Catalysis, Department of Chemistry, Zhejiang University, Hangzhou 310028, P. R. China.*

Correspondence: [maoshanjun@zju.edu.cn](mailto:maoshanjun@zju.edu.cn), [chemwy@zju.edu.cn](mailto:chemwy@zju.edu.cn).

# *Table of contents*

1. **Supplementary Methods**
   1. Synthesis of Pd nanoparticles
   2. Preparation of WO_3_
   3. Synthesis of Pd-based catalysts
   4. Preparation of Pd_1_/WO_3_ and Pd_1_/MoWO_3_
   5. Catalyst Characterizations
   6. Cycling stability test
   7. Selective poisoning experiment
2. **Supplementary Figures**

**Supplementary Figure 1.** Schematic illustration of the synthetic route to prepare Pd-based catalysts.

**Supplementary Figure 2.** TEM images of the PVP-protected 2.8 nm Pd particles and particle size distribution histograms.

**Supplementary Figure 3.** HRTEM images of the size-controlled Pd catalysts over different supports.

**Supplementary Figure 4.** FT-IR spectrum of the Pd/TiO_2_ after the removal of PVP.

**Supplementary Figure 5.** XRD patterns of those catalysts over various supports with the average Pd size of 2.8 nm.

**Supplementary Figure** **6.** Comparison for the activity of the MBY hydrogenation with and without hydrogen pretreatment

**Supplementary Figure 7.** Comparison for the selectivity versus conversion of the MBY hydrogenation with and without hydrogen pretreatment

**Supplementary Figure 8.** Images of the solution of Pd/WO_3_ and Pd/Al_2_O_3_ after different hydrogen pretreatment time.

**Supplementary Figure 9.** Morphology of the WO_3_ nanorods.

**Supplementary Figure 10.** Aberration-corrected HAADF-STEM image of the Pd/WO_3_.

**Supplementary Figure 11.** Morphology of the Pd/WO_3_ before and after hydrogen pretreatment.

**Supplementary Figure 12.** XRD patterns for fresh Pd/WO_3_ and activated Pd/WO_3_.

**Supplementary Figure 13.** Evolution of in situ H_2_ FT-IR spectra of Pd/WO_3_.

**Supplementary Figure** **14.** Evolution of in situ D_2_ FT-IR spectra of Pd/WO_3_。

**Supplementary Figure 15.** The catalytic performance and corresponding reaction rate of MBY semi-hydrogenation on Pd/WO_3_ after pre-activation in H_2_ for different period.

**Supplementary Figure 16.** Successive batch experiments of MBY semi-hydrogenation on Pd/WO_3_.

**Supplementary Figure 17.** XPS spectra of Pd 3*d* and W 4*f*. Raman spectra for fresh and activated Pd/WO_3_.

**Supplementary Figure 18.** Isotope effect in MBY semi-hydrogenation catalyzed by Pd/WO_3_-H.

**Supplementary Figure 19.** TEM images of the PVP-protected 4.6 nm Pd particles and particle size distribution histograms.

**Supplementary Figure 20.** TEM images of the PVP-protected 7.8 nm Pd particles and particle size distribution histograms.

**Supplementary Figure 21.** TEM image of the Pd/WO_3_ with different Pd particle sizes.

**Supplementary Figure 22.** XRD patterns of Pd/WO_3_ with different Pd particle sizes.

**Supplementary Figure 23.** EDX mapping images of different elements of Pd_1_/WO_3_.

**Supplementary Figure 24.** Normalized Pd K-edge X-ray absorption near-edge structure (XANES) spectra.

**Supplementary Figure 25.** EXAFS fitting curve of Pd_1_/WO_3_ at R space.

**Supplementary Figure 26.** XPS spectra of Pd 3*d* for fresh and activated Pd_1_/WO_3_.

**Supplementary Figure 27.** Conversion and TOF of the MBY hydrogenation with time on fresh and activated Pd_1_/WO_3_.

**Supplementary Figure 28.** The catalytic performance of H_2_ pretreated Pd/WO_3_ of different loadings of Pd.

**Supplementary Figure 29.** Specific activity of selective hydrogenation over Pd_1_/WO_3_ for different pretreated time.

**Supplementary Figure 30.** Conversion of MBY hydrogenation over 60 mg Pd/WO_3_ in the CO atmosphere and it was later switched to H_2_.

**Supplementary Figure 31.** Conversion of MBY hydrogenation over Pd/Al_2_O_3_ and Pd/WO_3_ after stirring with Li_2_SO_4_.

**Supplementary Figure 32.** The comparison of specific activity over Pd/WO_3_ and that physical mixed with WO_3_ after full activation.

**Supplementary Figure 33.** Selectivity to MBE as a function of MBY conversion over fresh and activated Pd/WO_3_ loaded with different Pd particle sizes.

**Supplementary Figure 34.** Conversion of the MBE hydrogenation with time on fresh and activated Pd_1_/WO_3_. Reaction condition: 10 mL of ethanol, 40 ℃, 1000 rpm, 1 mmol substrate, 0.3*10^-3^ wt% Pd.

**Supplementary Figure 35.** Contact angle measurements of Pd_1_/WO_3_ after hydrogen pretreatment for different period.

**Supplementary Figure 36.** Relationship between contact angle of Pd_1_/WO_3_ and selectivity after hydrogen pretreatment for different period.

**Supplementary Figure 37.** Conversion and selectivity of the MBY hydrogenation with time on Pd_1_/TiO_2_ and Pd_1_/Al_2_O_3_ before and after hydrogen pretreatment.

**Supplementary Figure 38.** Detail structures of Pd_1_/WO_3_ and Pd_1_/MoWO_3_ in the hydrogen spillover process.

**Supplementary Figure 39.** XRD patterns of Pd_1_/MoWO_3_, MoWO_3_ and WO_3_.

**Supplementary Figure 40.** Morphology of the MoWO_3_ nanorods.

**Supplementary Figure 41.** HAADF-STEM image and EDX mapping images of Pd_1_/MoWO_3_.

**Supplementary Figure 42.** Photos of as-synthesized WO_3_ and MoWO_3_.

**Supplementary Figure 43.** EPR spectra of WO_3_ and MoWO_3_.

**Supplementary Figure 44.** XPS spectra of Pd 3*d* for Pd_1_/WO_3_ and Pd_1_/MoWO_3_.

**Supplementary Figure 45.** Raman spectra and 1H solid-state NMR of Pd_1_/MoWO_3_.

**Supplementary Figure 46.** Evolution of in situ FT-IR spectra of H_2_ adsorbed on Pd_1_/WO_3_ and Pd_1_/MoWO_3_.

**Supplementary Figure 47.** Configurations and the calculated Eads values of MBY and MBE adsorption on Pd_1_/MoWO_3_ and Pd_1_/MoWO_3_-H.

**Supplementary Figure 48.** Temperature-programmed desorption experiments of C_2_H_2_ and C_2_H_4_ over Pd_1_/MoWO_3_ and Pd_1_/MoWO_3_-H

**Supplementary Figure 49.** Specific activity of selective hydrogenation over Pd_1_/MoWO_3_ for different pretreated time

**Supplementary Figure 50.** Comparison of specific activity and MBE selectivity for different catalysts.

**Supplementary Figure 51.** Catalytic activity and selectivity for the selective hydrogenation of MBY over commercial Lindlar catalyst.

**Supplementary Figure 52.** Comparison of each cycle in terms of conversion versu time curves on Pd_1_/MoWO_3_.

**Supplementary Figure 53.** Hydrogenation activity of different substrates.

**Supplementary Figure 54.** Solvent-free experiments over Pd_1_/MoWO_3_.

**Supplementary Figure 55.** Aberration-corrected HAADF-STEM image of Pt_1_/MoWO_3_, with single-site Pd marked by red circles.

**Supplementary Figure 56.** EDX mapping images of different elements of Pt_1_/MoWO_3_.

**Supplementary Figure 57.** XRD pattern of Pt_1_/MoWO_3_.

1. **Supplementary Tables**

**Supplementary Table 1.** EXAFS fitting parameters at the Pd K-edge for samples.

**Supplementary Table 2.** Catalytic performance of different catalysts for selective hydrogenation of MBY.

**Supplementary Table 3.** Catalytic performance of Pd_1_/MoWO_3_ for a series of alkynes.

**Supplementary Table 4.** Selective hydrogenation of nitrobenzene with different substitutions.

**Supplementary Table 5.** ICP results of the Pd loading for different catalysts.

**Supplementary Table 6.** The loadings of Pd of single atom catalysts and catalyst with the average Pd size of 2.8 nm, 4.6 nm and 7.8 nm estimated using an ICP spectrometer.

1. **Supplementary References**

# Supplementary Methods

## 1.1 Synthesis of Pd nanoparticles

The average size of Pd nanoparticles of 2.8 nm, 4.6 nm, and 7.8 nm were synthesized by modified colloidal methods. To synthesize 2.8 nm nanoparticles, a solution containing 60 mg PdCl_2_ and 2 g PVP in 30 ml ethylene glycol was heated to 165 °C for 30 min under Ar atmosphere. After cooling, 150 ml acetone was added to the dark brown solution to precipitate the particles, which were subsequently washed three times with acetone and dissolved in 36 ml ethanol to obtain a Pd concentration of 1 mg·ml^-1^. Similar methods were used to synthesized 4.6 nm and 7.8 nm Pd nanoparticles, with the mass ratio of PdCl_2_ and PVP changed to 1:10 and 1:5, respectively.

## 1.2 Preparation of WO_3_

WO_3_ support was synthesized using a simple hydrothermal method. Firstly, 3.4 g of ammonium metatungstate was dissolved in 40 ml deionized water, followed by the addition of 0.64 g of citric acid in 20 ml deionized water. The mixture was continuously stirring for 30 min and then transferred into an 80 ml Teflon-lined autoclave, where it was heated at 180 °C for 12 h. After natural cooling to room temperature, the blue precipitate was washed with distilled water and dried in vacuum at 70 °C for 12 h. The synthesis of MoWO_3_ followed a similar method, while for 3% Mo-doped WO_3_, 3.4 g ammonium metatungstate and 137 mg ammonium molybdate were initially dissolved in 40 ml of deionized water. The subsequent process was the same as for WO_3_ synthesis. The resulting products were designated as MoWO_3_.

## 1.3 Synthesis of Pd-based catalysts

In a typical procedure, 300 mg of supports were dissolved in 20 ml of DMF for 30 min. A specific quantity of as-synthesized Pd NP (nanoparticle) solution (the theoretical loading of Pd was 0.3 wt%) was added under vigorous stirring. The mixture was stirred at room temperature for 3 h. Subsequently, PVP-Pd/support catalyst was collected via centrifugation for 10 min and washed with acetone. The capping agent, PVP, was removed from the surface of the catalysts via a calcination method. The samples were calcined in static air at 300 °C for 1 h (with a heating rate of 5 °C min^-1^). The resulting yellow powder was then reduced in a pure H_2_ atmosphere at 150 °C for 1 h using a heating rate of 2 °C min^-1^ to yield Pd-based catalysts.

## 1.4 Preparation of Pd_1_/WO_3_ and Pd_1_/MoWO_3_

The Pd_1_/WO_3_ catalysts were synthesized via the wetness impregnation method. Specifically, 300 mg of WO_3_ was dispersed in 15 ml of deionized water and stirred for 30 min. Simultaneously, 273 mg of NH_4_HCO_3_ was dissolved in 20 ml of deionized water, followed by sonication for 30 min. The resulting solutions were then combined and stirred for an additional 30 min, resulting in a flocculent blue precipitate. Next, 100 mg PdCl_2_ was first dissolved in 10 ml hydrochloric acid solution (0.6 M) to obtain PdCl_2_ solution. 150 μL PdCl_2_ hydrochloric acid solution (containing 6 mg·ml^-1^ Pd) diluted with 8 ml deionized water was added in the WO_3_ dispersion and constantly stirred for 1 h. The resulting product was separated via centrifugation and subsequently dried under vacuum at 70 ºC. Finally, the catalysts were reduced in H_2_ (flow rate=50 ml·min^-1^, heating rate=2 ºC·min^-1^) at 150 °C for 1 h, yielding 0.3% Pd_1_/WO_3_. The Pd/MoWO_3_ catalysts were prepared using the same protocol, with MoWO_3_ was used as the support material. The loading amount of Pd was regulated by the volume of the precursor solution.

## 1.5 Catalyst Characterizations

Solid state NMR measurements were performed on Bruker Avance III HD 400 MHz spectrometers using 3.2 mm magic-angle spinning probes. Samples were packed in 3.2 mm rotors in an argon filled glove box and were spun at 15 kHz during measurements.

Pd *K*-edge analysis was performed with Si (311) crystal monochromators at the BL14W1 beamlines at the Shanghai Synchrotron Radiation Facility (SSRF) (Shanghai, China). Before the analysis at the beamline, samples were pressed into thin sheets with 1 cm in diameter and sealed using Kapton tape film. The XAFS spectra were recorded at room temperature using a 4-channel Silicon Drift Detector (SDD) Bruker 5040. Pd K-edge extended X-ray absorption fine structure (EXAFS) spectra were recorded in fluorescence mode. The XAFS spectra of these standard samples (Pd foil and PdO) were recorded in transmission mode.

The spectra were processed and analyzed by the software codes Athena and Artemis. Temperature programmed desorption (TPD) experiments were conducted with a TCD using 10 vol% C_2_H_2_ or C_2_H_4_ in Ar, respectively. The sample (100 mg) was pretreated by He flow at 150 ºC for 1 h with a heating rate of 5 °C min^-1^. When the sample was cooled to 25 °C, C_2_H_2_ or C_2_H_4_ was introduced into the sample cell for 1 h and weakly adsorbed species were removed by purging with He for 1 h at the same temperature. Then the TPD experiments were conducted with a heating rate of 5 °C min^-1^.

In situ FTIR spectra of H_2_ adsorbed on solid samples were collected on a Bruker Vector 70. Before the tests, the as-synthesized catalysts were pretreated with N_2_ flow at 150 ºC for 1 h with a heating rate of 5 °C min^-1^ to remove the vapor and impurities. After the samples were cooled to room temperature, the background spectrum was recorded and H_2_ was then introduced into the sample cell with a flow rate of 20 ml·min^−1^. Then, IR spectra were recorded at constant intervals.

## 1.6 Cycling stability test

The 40 mg catalysts and 4 mmol substrate were mixed in a reaction mixture, which was then dispersed in 10 ml ethanol. The mixture was constantly stirred at 1000 rpm and 40 °C under 1 bar H_2_. The reaction was stopped at a fixed time, and the catalysts were removed using centrifugal separation. The used catalysts were washed with ethanol for 3 times and dried overnight in vacuum at 60 °C. Due to the loss of catalyst during centrifugation, parallel experiments were conducted to compensate for the waste during the recycling test, with consistent experimental conditions as before.

## 1.7 Selective poisoning experiment

To investigate the impact of support on the reaction, CO was utilized as a poisoning agent. Typically, a certain amount of catalysts was dispersed in 10 ml of ethanol, and a balloon filled with H_2_ (1 bar) was then connected to the flask and purged for several times to remove air for 2 h. CO was introduced into the flask after the H_2_ pretreatment to poison the Pd sites, and 2 h later, the substrates were added to the system. Samples were collected at regular intervals with a syringe to monitor the progress of the reaction. Reaction condition: 10 mL of ethanol, 40 ℃, 1 bar CO, and 1000 rpm. 0.25 mmol substrate, 0.028 mol% Pd. Additionally, Li_2_SO_4_ was used to poison the hydroxyls on the support surface. While other conditions remained unchanged, 300 mg of Li_2_SO_4_ was added to the system immediately after H_2_ pretreatment and stirred for 12 h. Then, 1 mmol substrates and H_2_ were sequentially introduced into the system. Product distribution was analyzed using GC-FID.

# Supplementary Figures

## **
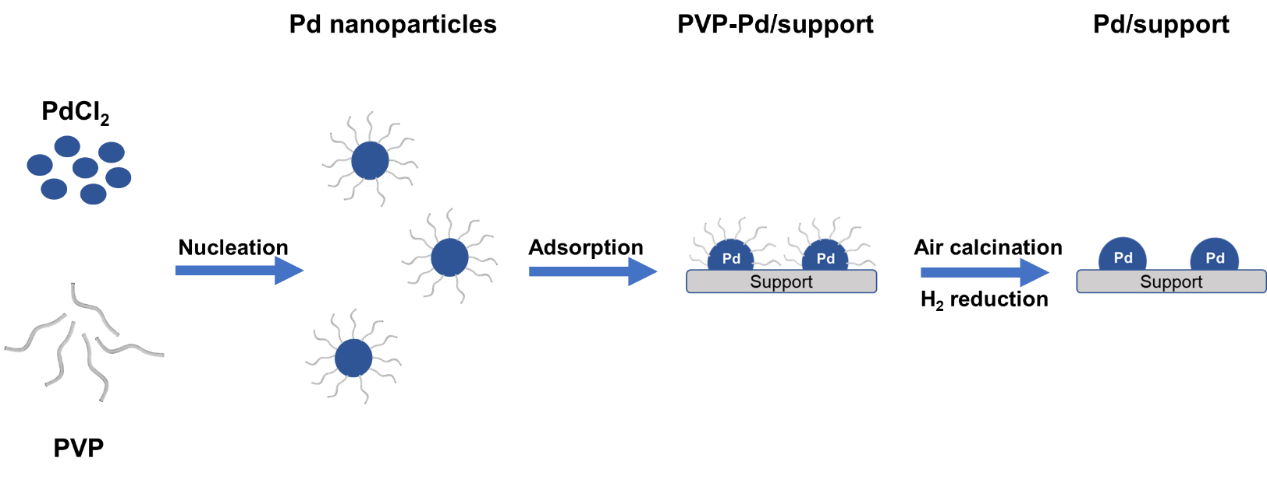
Supplementary Figure 1.** Schematic illustration of the synthetic route to prepare Pd-based catalysts.


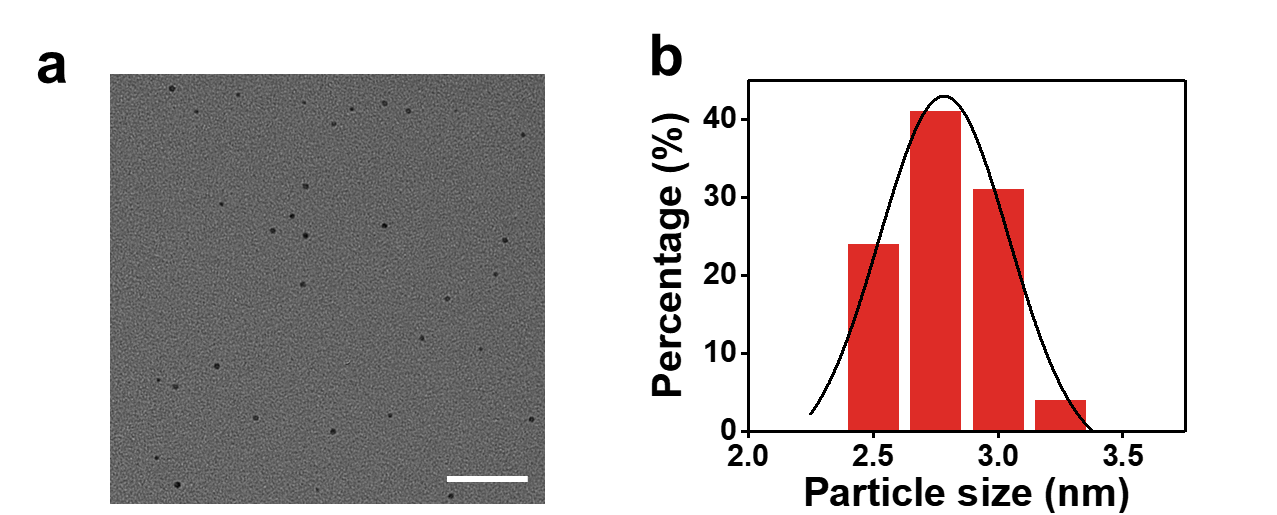


## **Supplementary Figure 2.** (a) TEM images of the PVP-protected 2.8 nm Pd particles. Scale bar: 50 nm. (b) Particle size distribution histograms.


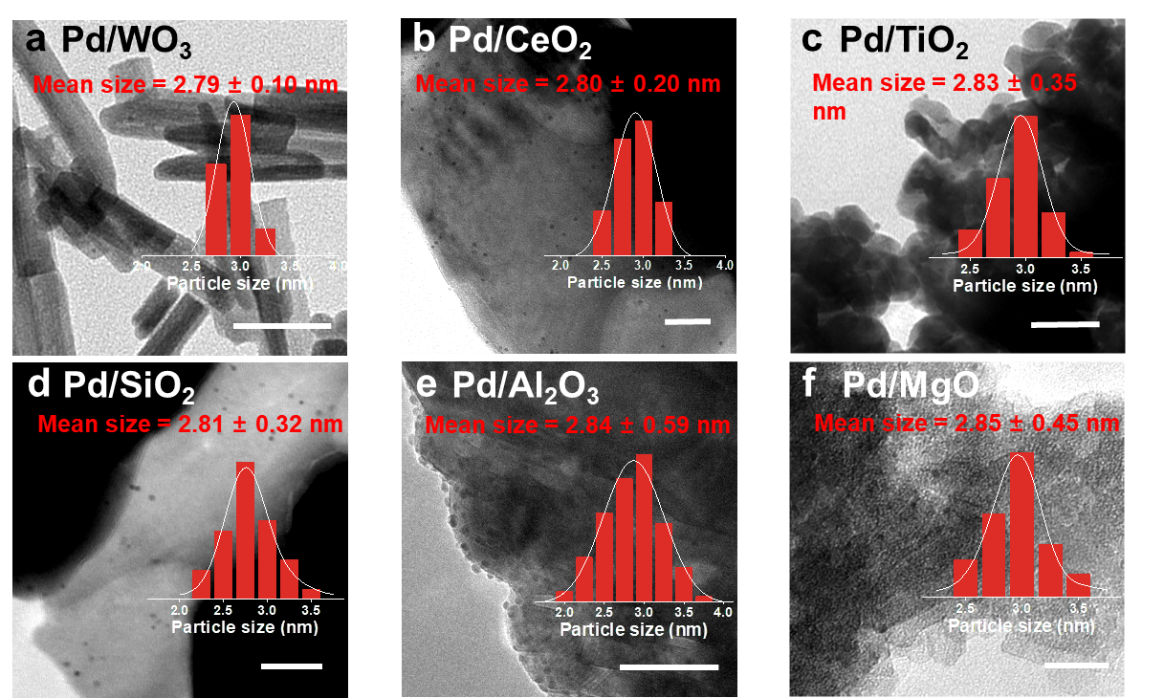


## **Supplementary Figure** 3. HRTEM images of the size-controlled Pd catalysts over different supports and the inset are their size distribution histograms; scale bar: 50 nm. (a) Pd/WO_3_, (b) Pd/CeO_2_, (c) Pd/TiO_2_, (d)Pd/SiO_2_, (e) Pd/Al_2_O_3_, (f) Pd/MgO.

## **Supplementary Figure** 4. FT-IR spectrum of the Pd/TiO_2_ before and after the removal of PVP. The absence of the peak of C-H stretching vibration belonging to PVP confirmed that the residual PVP was removed thoroughly by calcination^1^.


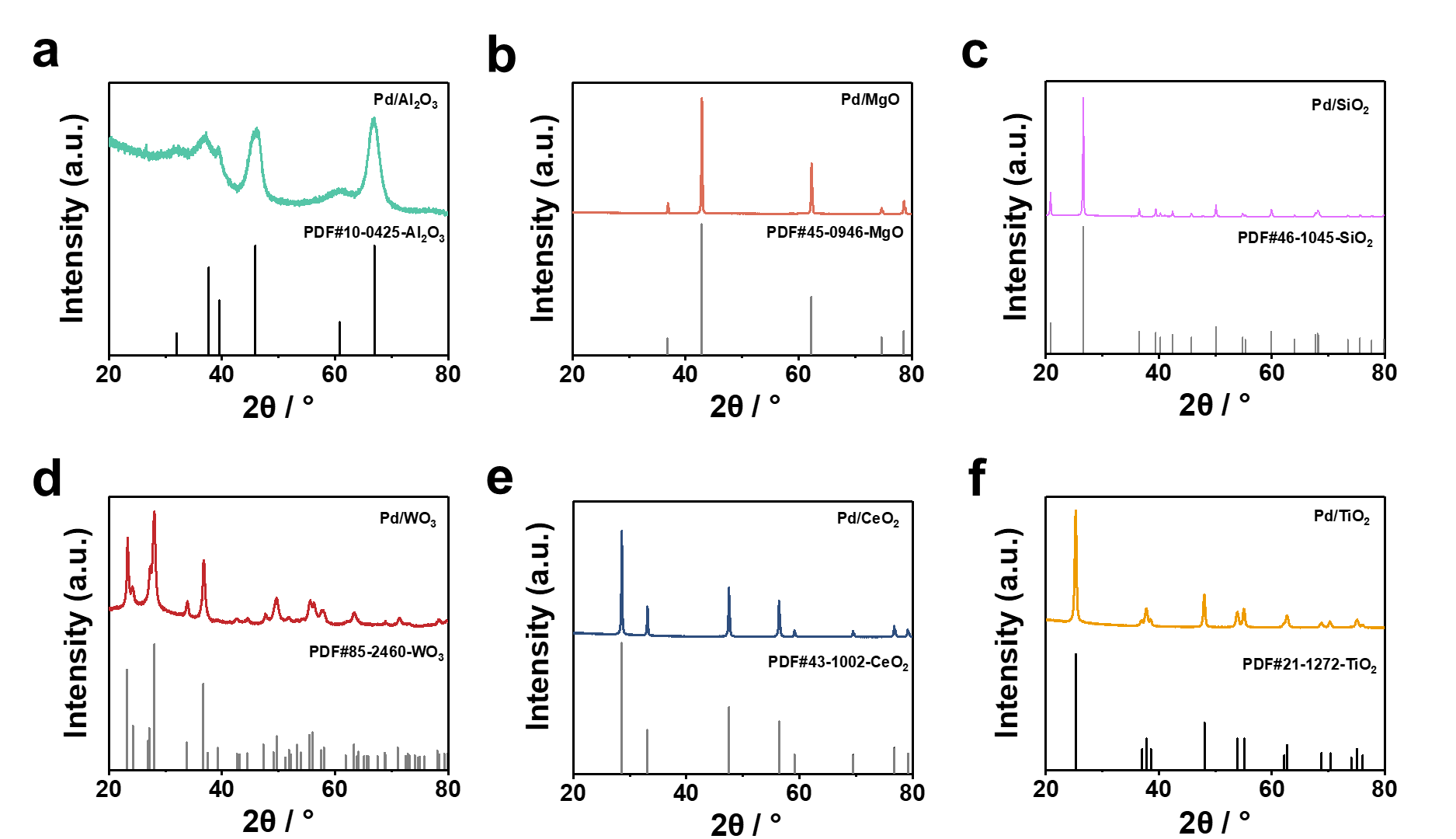


## **Supplementary Figure** 5. XRD patterns of those catalysts over various supports with the average Pd size of 2.8 nm. (a) Pd/Al_2_O_3_, (b) Pd/MgO, (c) Pd/SiO_2_, (d) Pd/WO_3_, (e) Pd/CeO_2_, (f) Pd/TiO_2_.


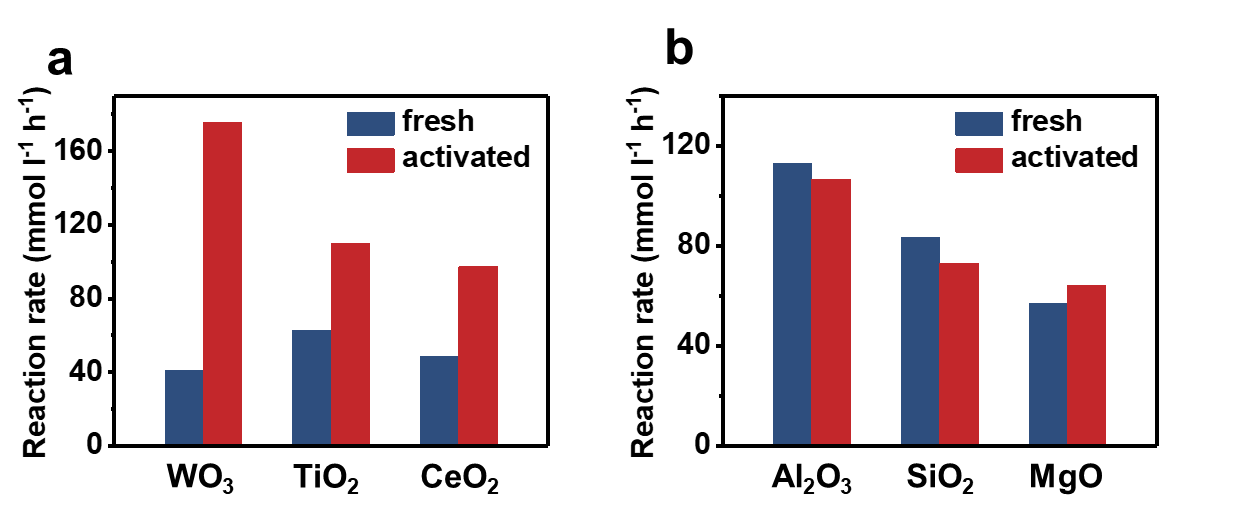


## **Supplementary Figure** 6. Comparison for the activity of the MBY hydrogenation with and without hydrogen pretreatment over reducible supports (a) and irreducible supports (b).


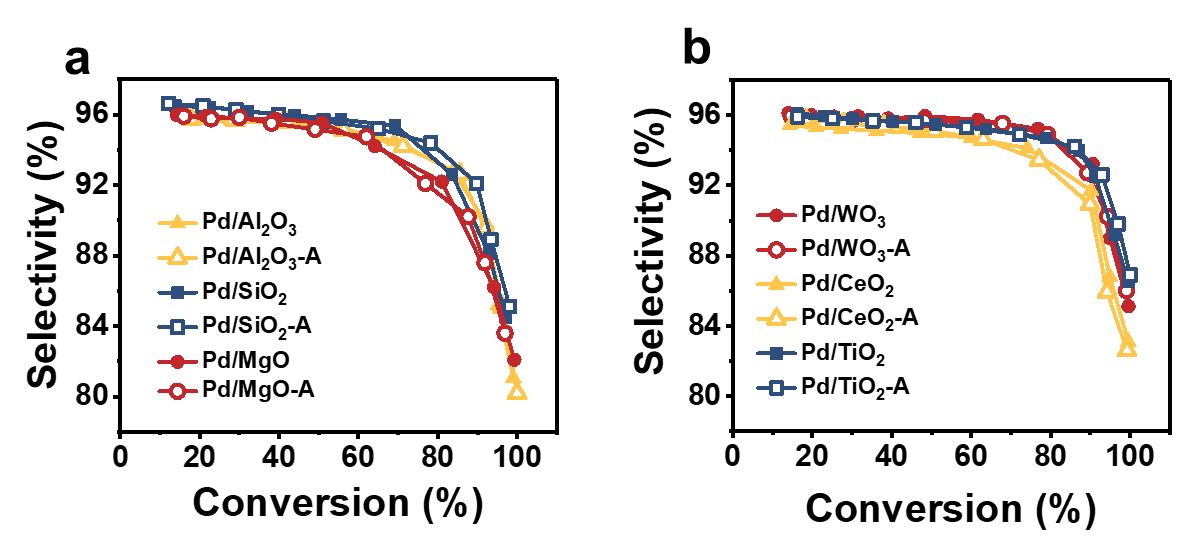


## **Supplementary Figure** 7. Comparison for the selectivity versus conversion of the MBY hydrogenation with and without hydrogen pretreatment over reducible supports (a) and irreducible supports (b).

**
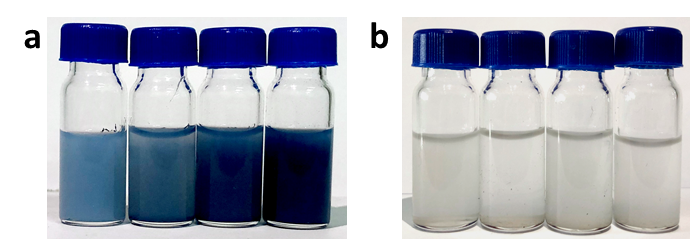
**

## **Supplementary Figure** 8. Images of the reaction solution of (a) Pd/WO_3_ and (b) Pd/Al_2_O_3_ after different hydrogen pretreatment time with 0 min, 10 min, 20 min and 30 min from left to right. Each vessel contains 0.5 ml reaction solution.


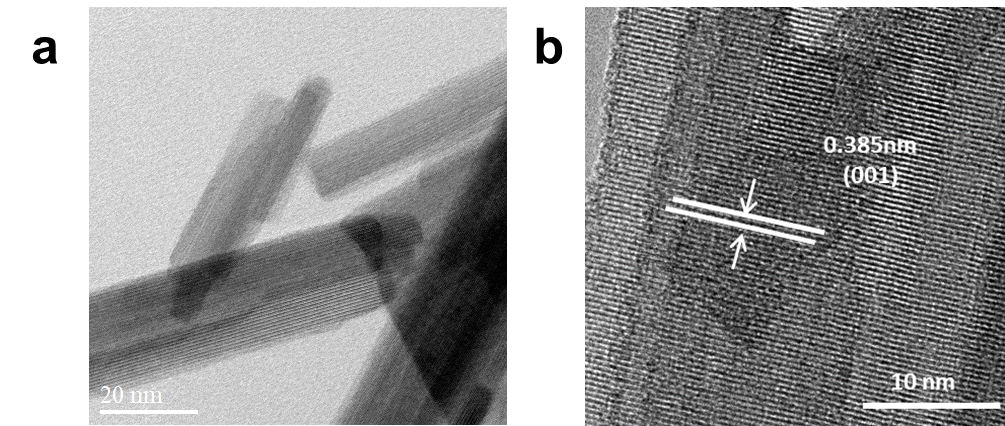


## **Supplementary Figure** 9. Morphology of the WO_3_ nanorods. (a) TEM of WO_3_. (b) HRTEM of WO_3_ and corresponding lattice spacing.


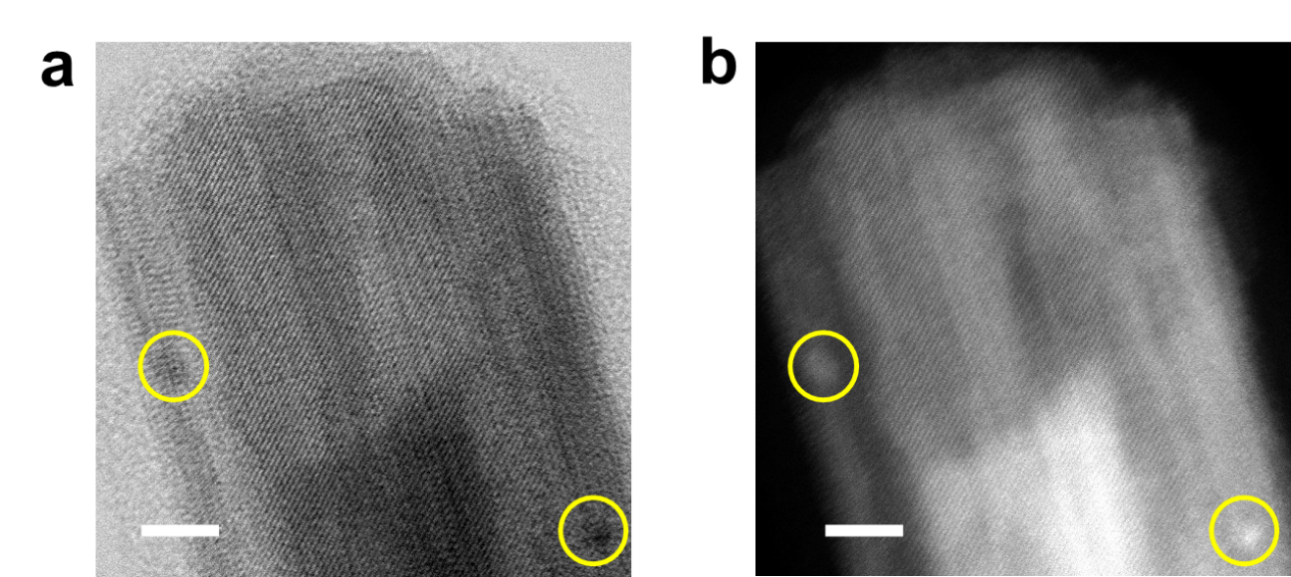


## **Supplementary Figure** 10. Aberration-corrected HAADF-STEM image of the Pd/WO_3_. Scale bar: 5 nm.


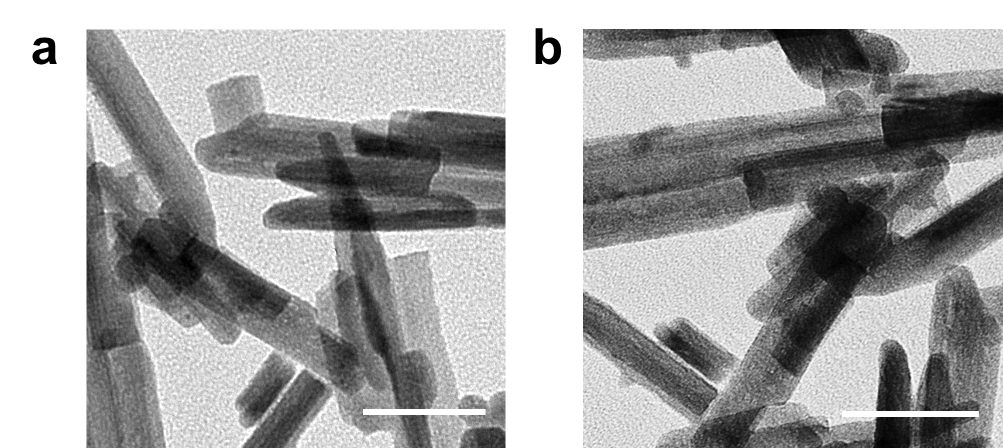


## **Supplementary Figure** 11. Morphology of the Pd/WO_3_ before (a) and after (b) hydrogen pretreatment. Scale bar: 100 nm.

## **Supplementary Figure** 12. XRD patterns for fresh Pd/WO_3_ and activated Pd/WO_3_.

## **Supplementary Figure** 13. Evolution of in situ FT-IR spectra of Pd/WO_3_ in a flow of H_2_.


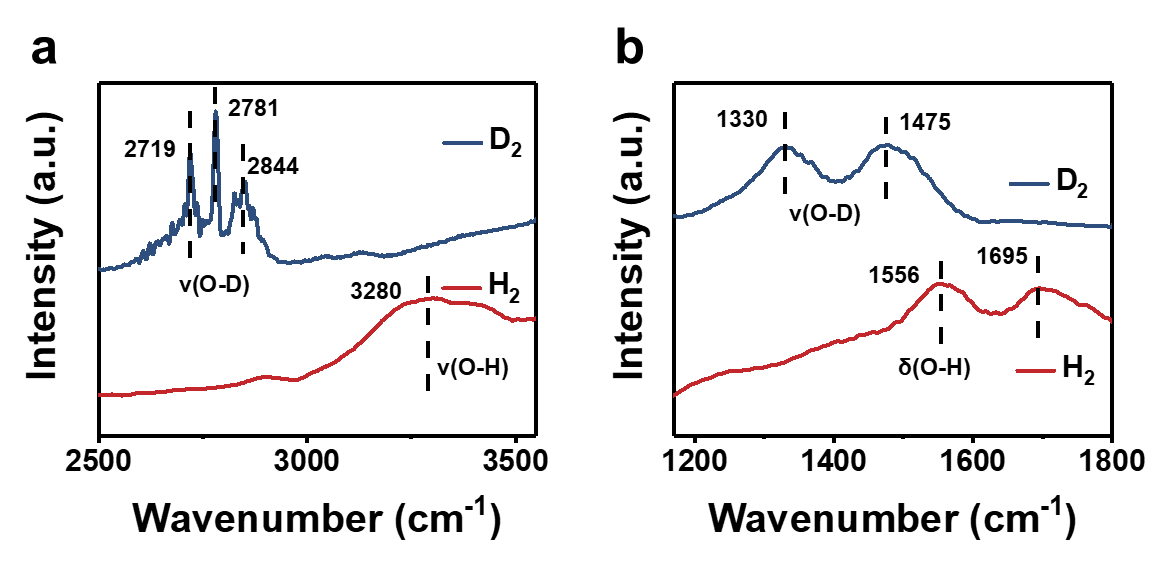


## **Supplementary Figure** 14. Evolution of in situ FT-IR spectra of Pd/WO_3_ (2.8 nm) in a flow of D_2_ and H_2_. (a) FT-IR spectra at wavelength of 2500 cm^-1^-3500 cm^-1^; (b) FT-IR spectra at wavelength of 1200 cm^-1^-1800 cm^-1^.


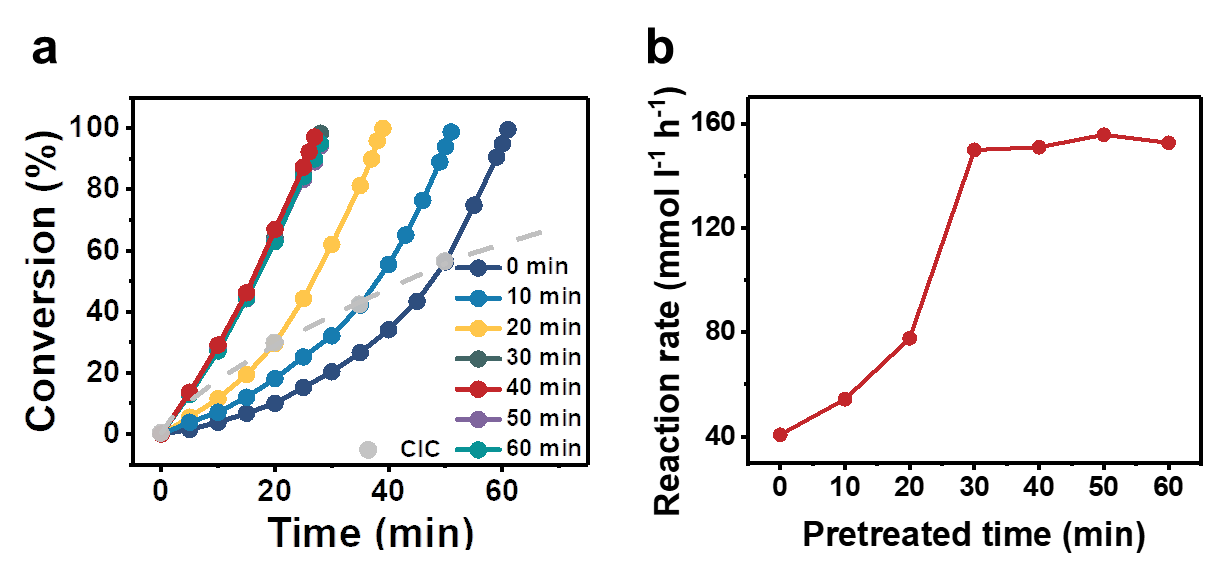


## Supplementary **Figure** 15. (a) The catalytic performance and (b) corresponding reaction rate of MBY semi-hydrogenation on Pd/WO_3_ (2.8 nm) after pre-activation in H_2_ for different period. Reaction condition: 10 ml of ethanol, 40 ℃, 1 bar H_2_, and 1000 rpm. 1 mmol substrate, 10 mg catalyst. Regarding the relationship between catalyst activation and kinetics, the concentration of substrates at the inflection point of the kinetic behaviour can be regarded as the critical inhibitory concentration (CIC), beyond which the hydrogenation process would be restrained by the competitive adsorption. With the extension of the pretreatment time, CIC gradually reduced, together with the decreased reaction order of MBY.

## Supplementary Figure 16. Successive batch experiments of MBY semi-hydrogenation on Pd/WO_3_ (2.8 nm). Reaction condition: 10 ml of ethanol, 40 ℃, 1 bar H_2_, and 1000 rpm. 1 mmol substrate, 10 mg catalyst.


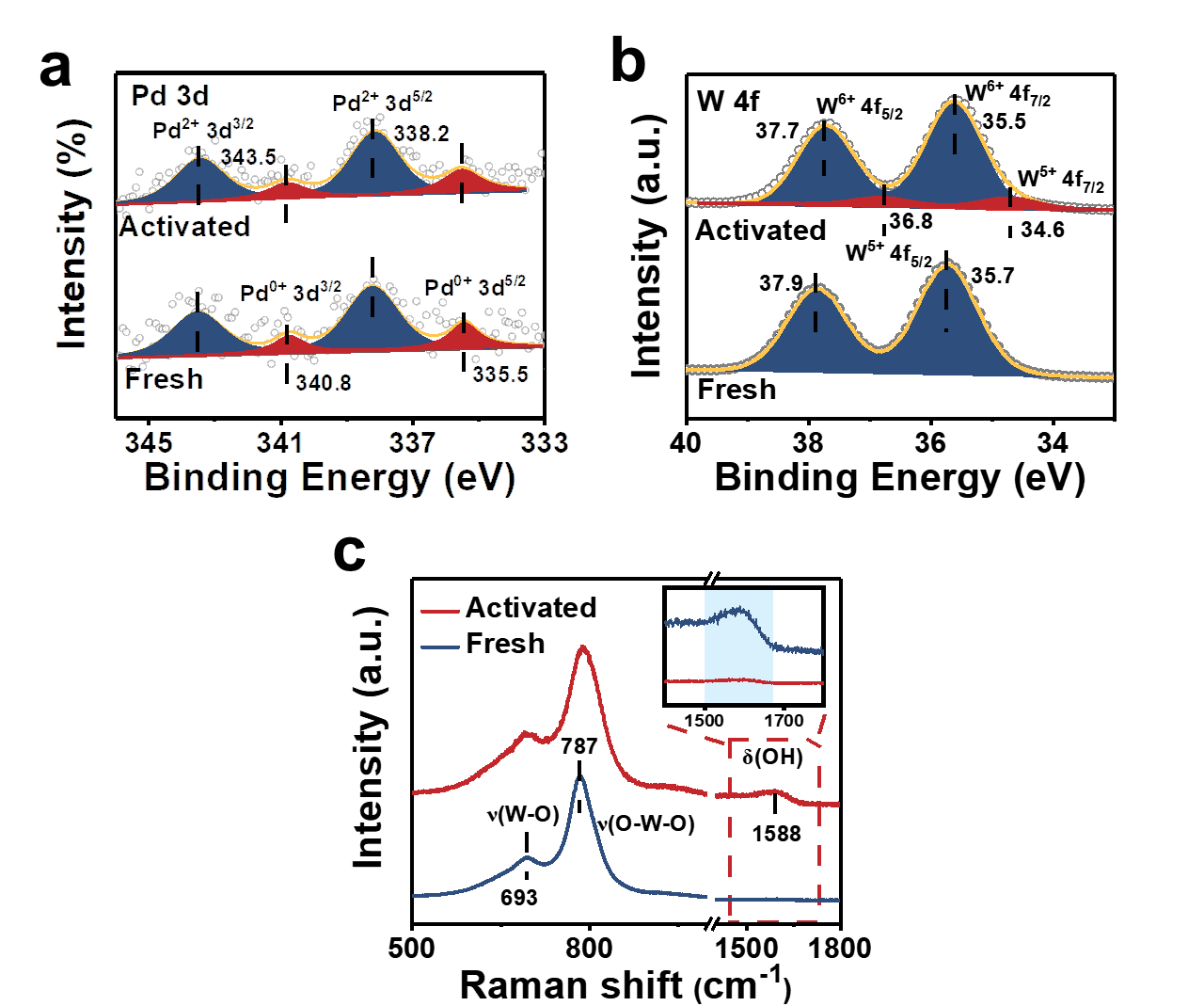


## Supplementary Figure 17. (a-b) High-resolution XPS spectra of Pd 3*d* (a) and W 4*f* (b). (c) Raman spectra for fresh and activated Pd/WO_3_.

## Supplementary Figure 18. Isotope effect in MBY semi-hydrogenation catalyzed by Pd/WO_3_-H, showing a typical secondary isotope effect.


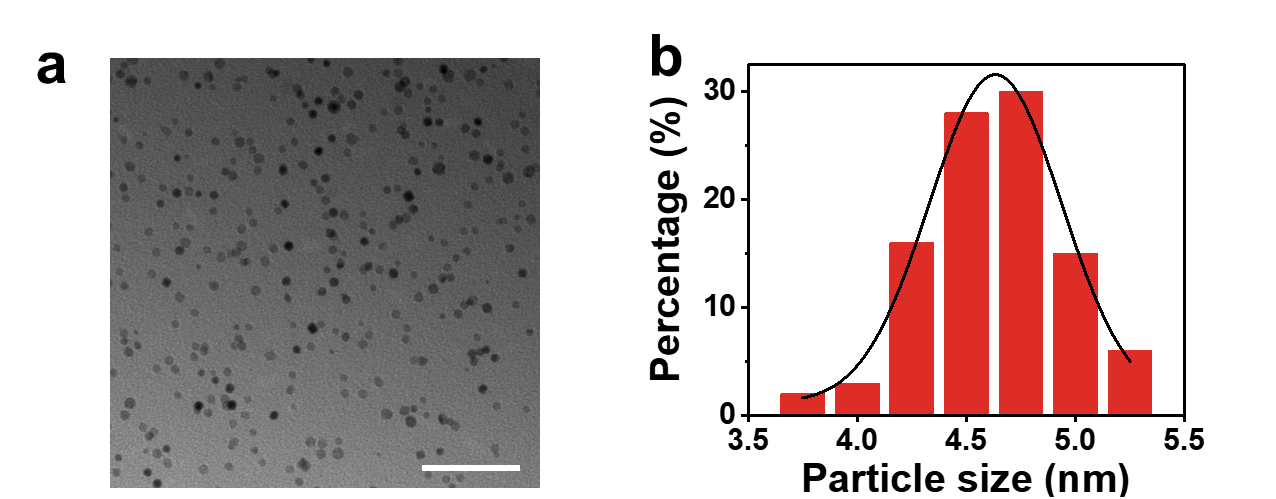


## Supplementary Figure 19. (a) TEM images of the PVP-protected 4.6 nm Pd particles. Scale bar: 50 nm. (b) Particle size distribution histograms.


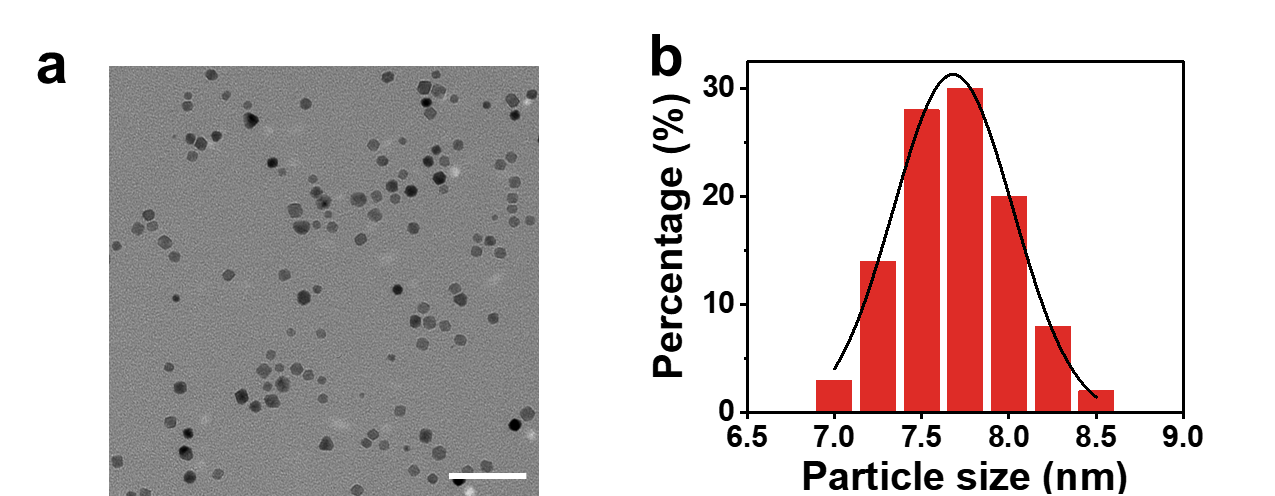


## Supplementary Figure 20. (a) TEM images of the PVP-protected 7.8 nm Pd particles. Scale bar: 50 nm. (b) Particle size distribution histograms.


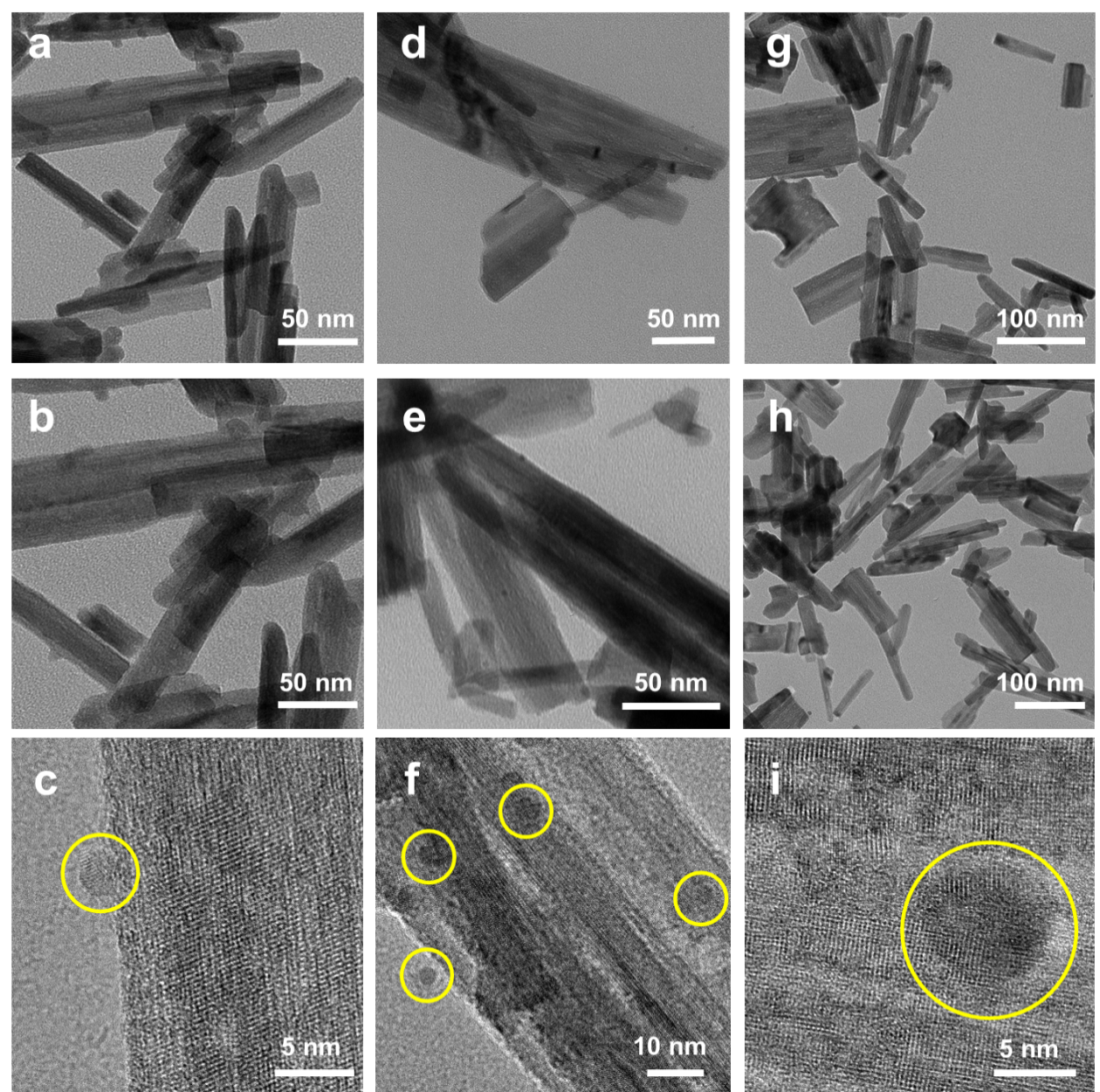


## Supplementary Figure 21. TEM image of the 0.3% Pd/WO_3_ with different Pd particle sizes: (a, b, c) 2.8 nm, (d, e, f) 4.6 nm, (g, h, i) 7.8 nm.

__

## Supplementary Figure 22. XRD patterns of as-synthesized Pd_1_/WO_3_, 2.8 nm Pd/WO_3_, 4.6 nm Pd/WO_3_ and 7.8 nm Pd/WO_3_.

_
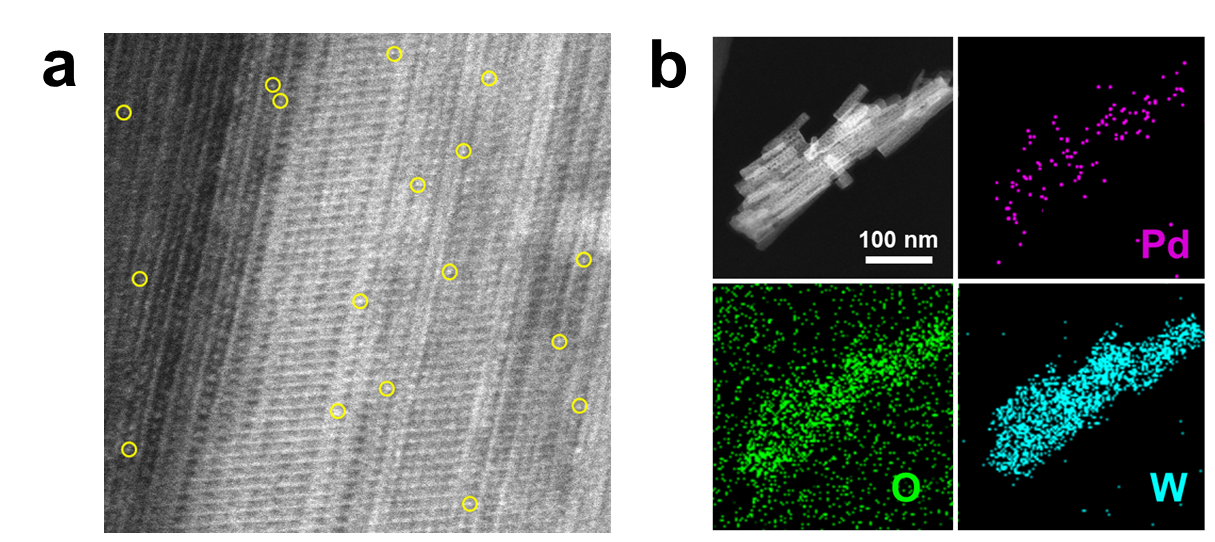
_

## Supplementary Figure 23. (a) Aberration-corrected HAADF-STEM image of Pd_1_/WO_3_, (b) EDX mapping images of different elements of Pd_1_/WO_3_.

## Supplementary Figure 24. Normalized Pd K-edge X-ray absorption near-edge structure (XANES) spectra (inset: expansion of the highlighted region)

## Supplementary Figure 25. EXAFS fitting curve of Pd_1_/WO_3_ at R space.

## Supplementary Figure 26. XPS spectra of Pd 3*d* for fresh and activated Pd_1_/WO_3_.


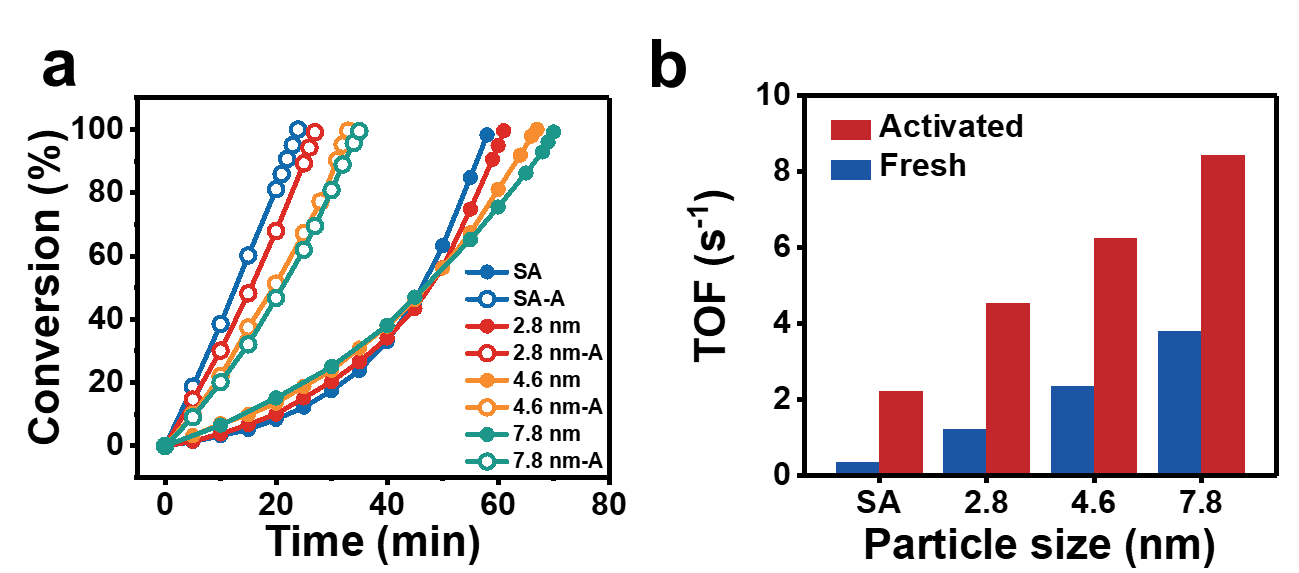


## Supplementary Figure 27. (a) Conversion of the MBY hydrogenation with time on fresh and activated Pd/WO_3_ loaded different size-controlled Pd NPs and single atoms. (b) TOF of the MBY hydrogenation on fresh and activated Pd_1_/WO_3_ loaded different size-controlled Pd NPs and single atoms (TOF based on the Pd sites).


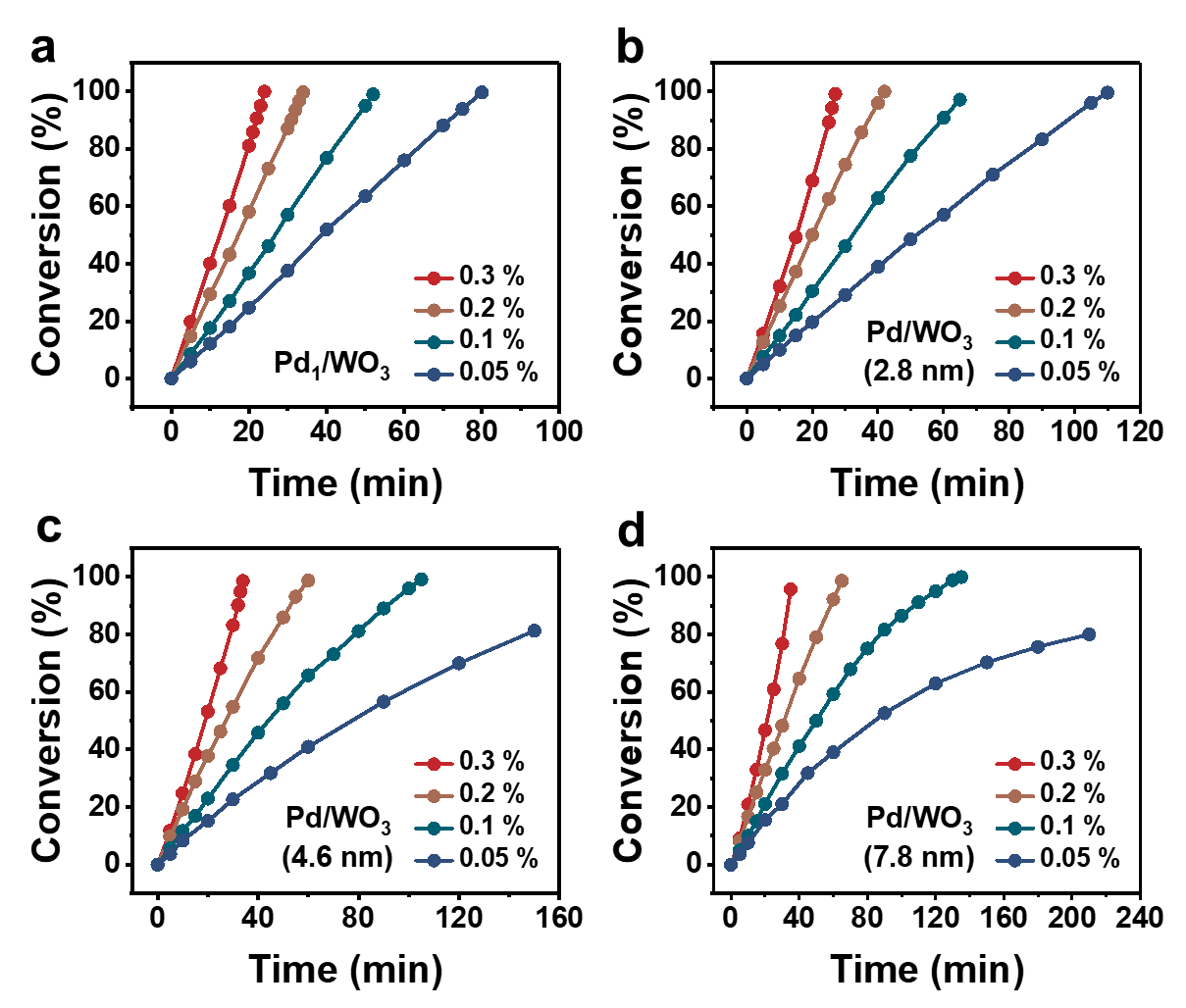


## Supplementary Figure 28. The catalytic performance of H_2_ pretreated Pd/WO_3_ of different loadings of Pd: a. Pd_1_/WO_3_; b, Pd/WO_3_ (2.8 nm); c, Pd/WO_3_ (4.6 nm); d, Pd/WO_3_ (7.6 nm).

## Supplementary Figure 29. Specific activity of selective hydrogenation over Pd_1_/WO_3_ for different pretreated time.


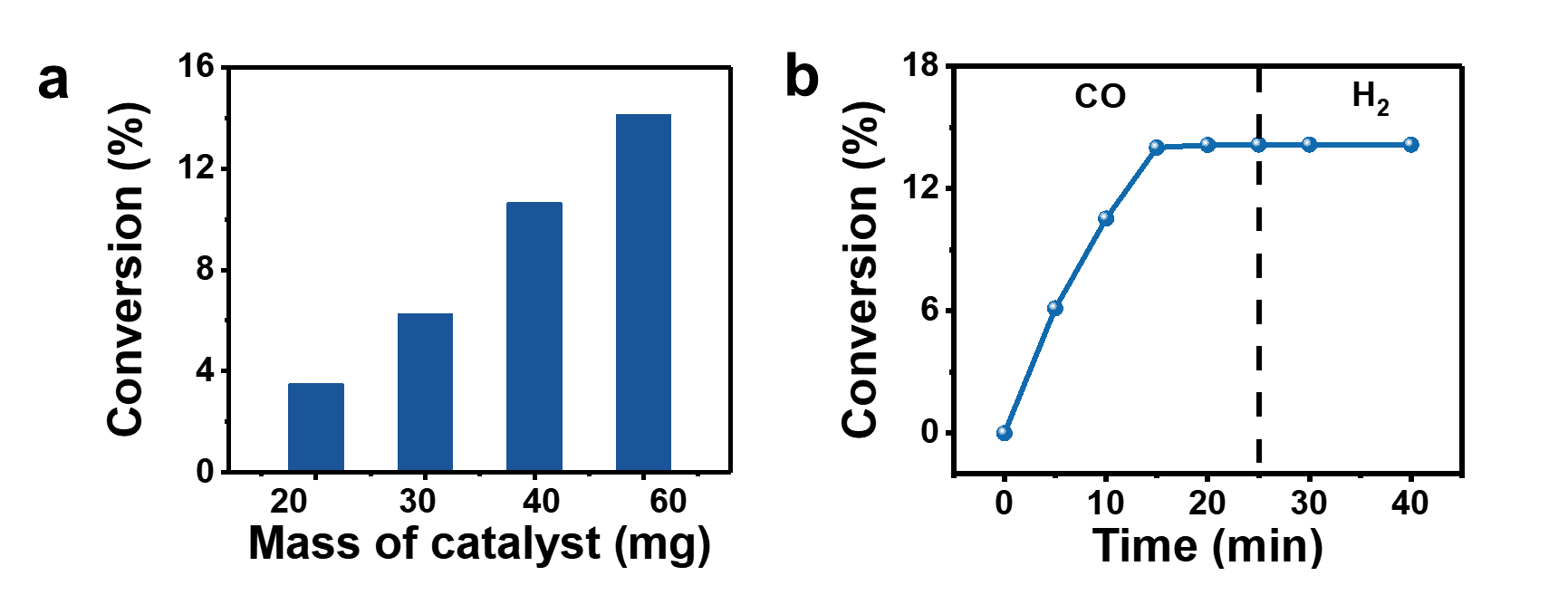


## Supplementary Figure 30. (a), Conversion of MBY hydrogenation in the CO atmosphere toward different loading of Pd in the premise of controlling the total amount of Pd to be consistent. (b), Conversion of MBY hydrogenation over 60 mg Pd/WO_3_ (2.8 nm) in the CO atmosphere and it was later switched to H_2_. Reaction condition: 10 mL of ethanol, 40 ℃, 1 bar H2, and 1000 rpm. 0.25 mmol substrate, 0.028 mol % Pd. No further hydrogenation occurred when the atmosphere was switched to H_2_, i.e., the Pd sites were completely deactivated by CO and the remained activity was attributed to the spillover hydrogen.


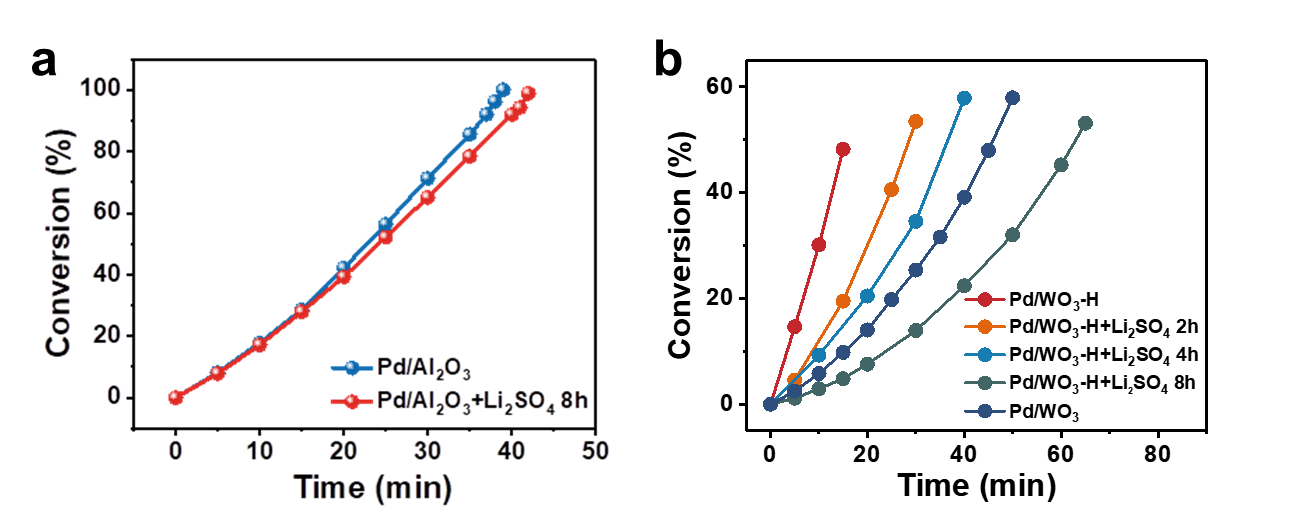


## Supplementary Figure 31. (a) Conversion of MBY hydrogenation over Pd/Al_2_O_3_ (2.8 nm) after stirring with Li_2_SO_4_ for 8 h. (b) Conversion of MBY hydrogenation over Pd/WO_3_ (2.8 nm) after stirring with Li_2_SO_4_ for various periods compared with no addition. Reaction condition: 10 mL of ethanol, 40 ℃, 1 bar H_2_, and 1000 rpm. 0.1 mmol substrate, 0.028 mol % Pd, 200 mg Li_2_SO_4_. The slightly decreased activity of Pd/Al_2_O_3_ after Li_2_SO_4_ treatment for 8 h indicated that the activity of Pd sites were not affected in general, confirming the preferential poisoning of OH groups by Li^+^.

## Supplementary Figure 32. The comparison of specific activity over Pd/WO_3_ and that physical mixed with WO_3_ after full activation. 0.1% Pd/WO_3_ performed better than the mixed 0.3% Pd/WO_3_ + WO_3_ combination on the premise of the same amount of Pd and WO_3_ support, which was attributed to the easier migration of hydrogen derived from the uniform dispersion of Pd nanoparticles.


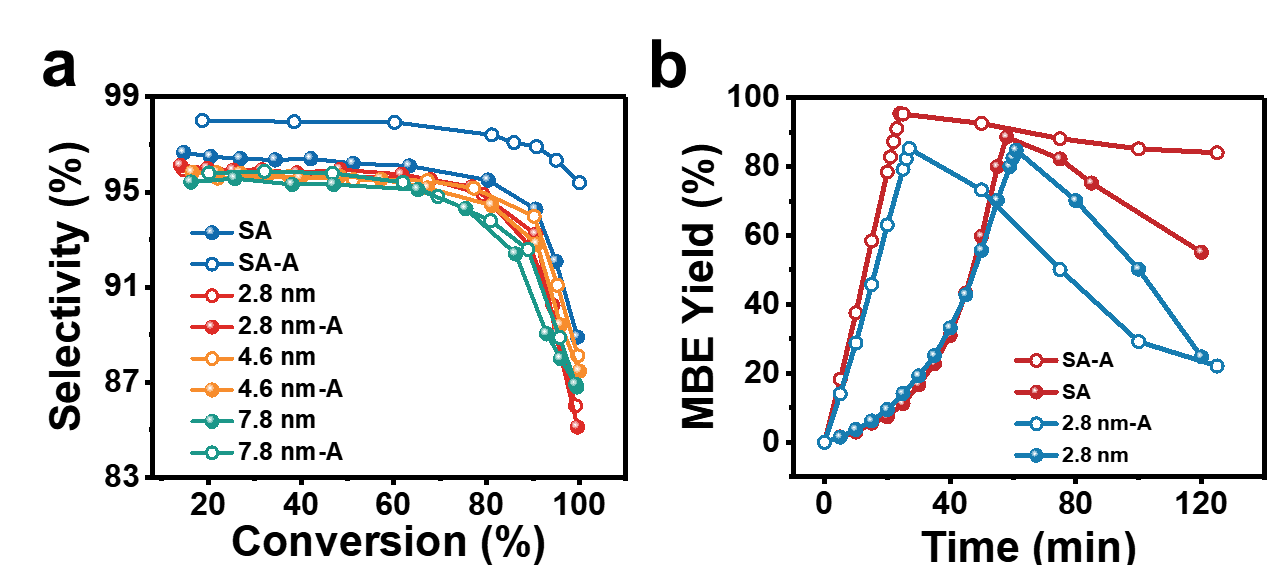


## Supplementary Figure 33. (a) Selectivity to MBE as a function of MBY conversion over fresh and activated Pd/WO_3_ loaded with different size-controlled Pd NPs and single atoms. (b) MBE yield in the hydrogenation of MBY over Pd_1_/WO_3_ and Pd_1_/WO_3_-H, compared with the 2.8 nm Pd/WO_3_ and 2.8 nm Pd/WO_3_-H. Reaction condition: 10 mL of ethanol, 40 ℃, 1000 rpm, 1 mmol substrate, 0.3*10^-3^ wt% Pd.

## Supplementary Figure 34. Conversion of the MBE hydrogenation with time on fresh and activated Pd_1_/WO_3_. Reaction condition: 10 mL of ethanol, 40 ℃, 1000 rpm, 1 mmol substrate, 0.3*10^-3^ wt% Pd.

**
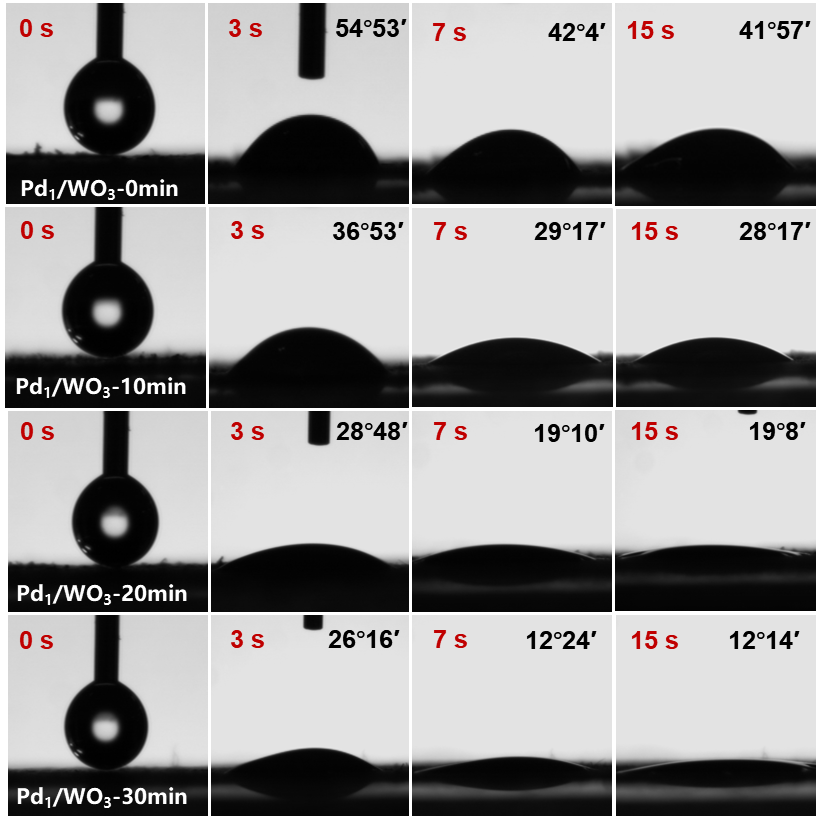
**

## Supplementary Figure 35. Contact angle measurements of Pd_1_/WO_3_ after hydrogen pretreatment for different period.

## Supplementary Figure 36. Relationship between contact angle of Pd_1_/WO_3_ and selectivity after hydrogen pretreatment for different period.


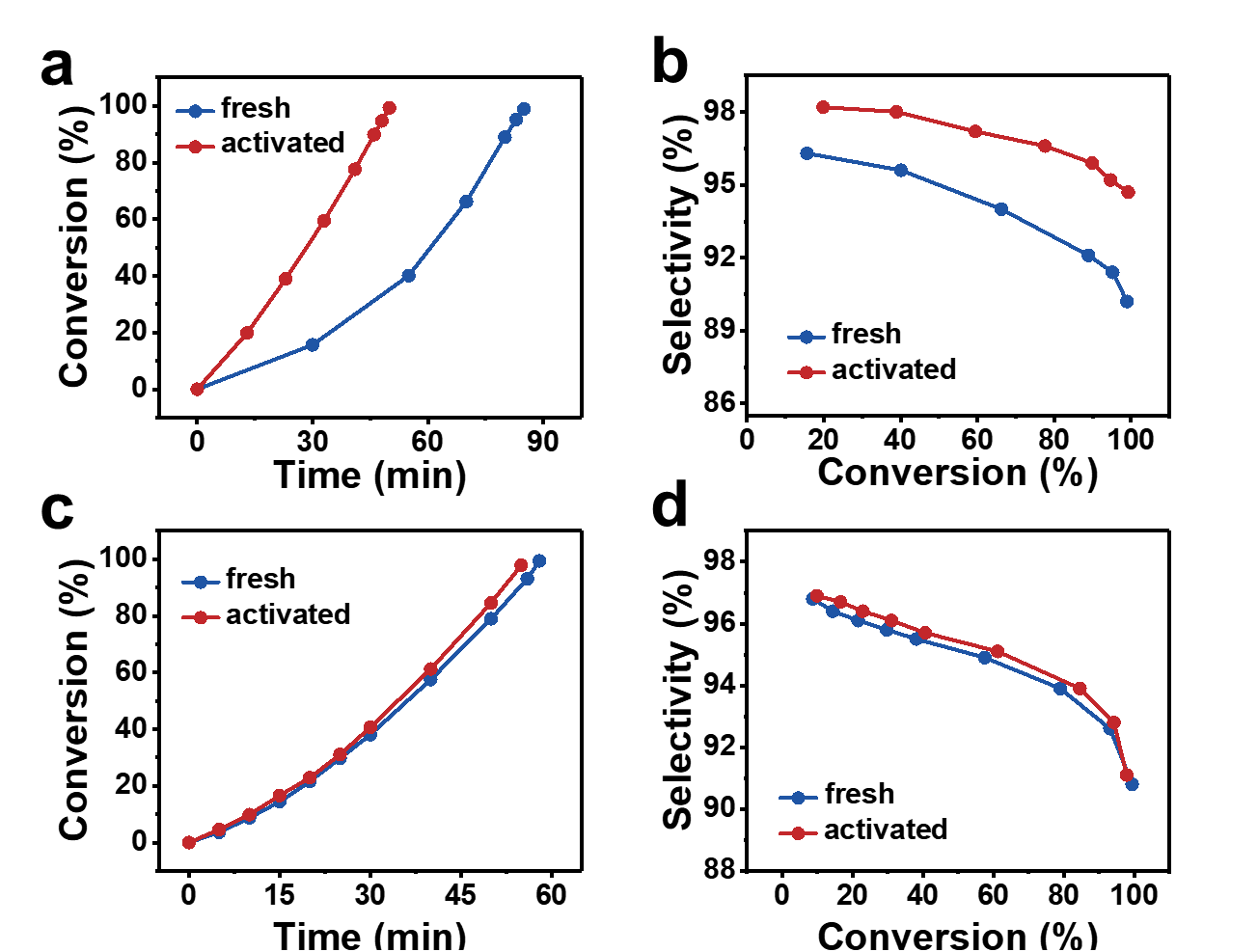


## Supplementary Figure 37. Conversion and selectivity of the MBY hydrogenation with time on Pd_1_/TiO_2_ (a-b) and Pd_1_/Al_2_O_3_ (c-d) before and after hydrogen pretreatment. The inferior apparent activity of Pd_1_/TiO_2_ compared with Pd_1_/WO_3_ could be attributed to the relatively lower capacity of hydrogen spillover.


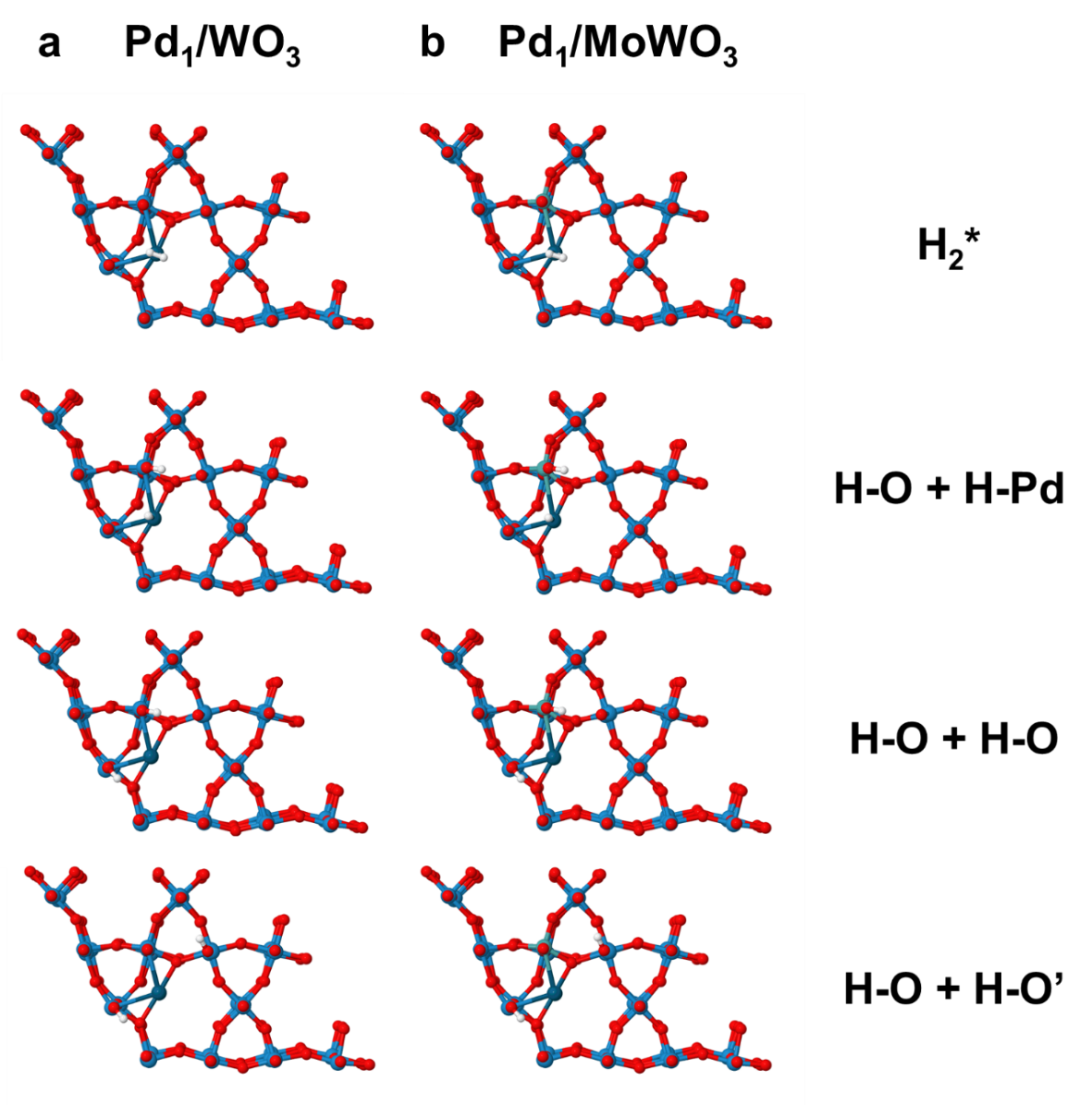


## Supplementary Figure 38. Detail structures of (a) Pd_1_/WO_3_ and (b) Pd_1_/MoWO_3_ in the hydrogen spillover process. Color code – dark blue: Pd, light blue: W, white: H, red: O, cyan: Mo.

## Supplementary Figure 39. XRD patterns of Pd_1_/MoWO_3_, MoWO_3_ and WO_3_.


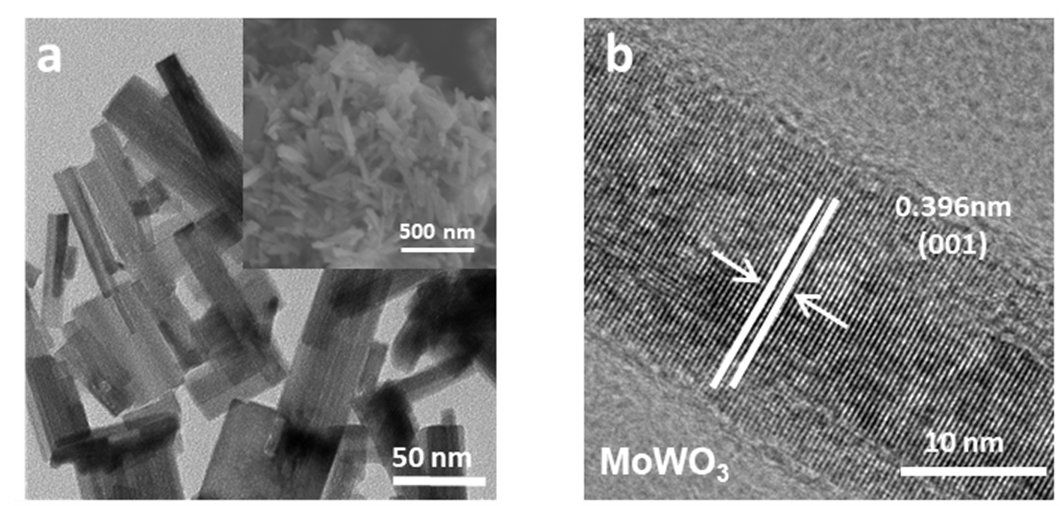


## Supplementary Figure 40. Morphology of the MoWO_3_ nanorods. (a) TEM and SEM (inset) of MoWO_3_. (b) HRTEM of MoWO_3_ and corresponding lattice spacing.


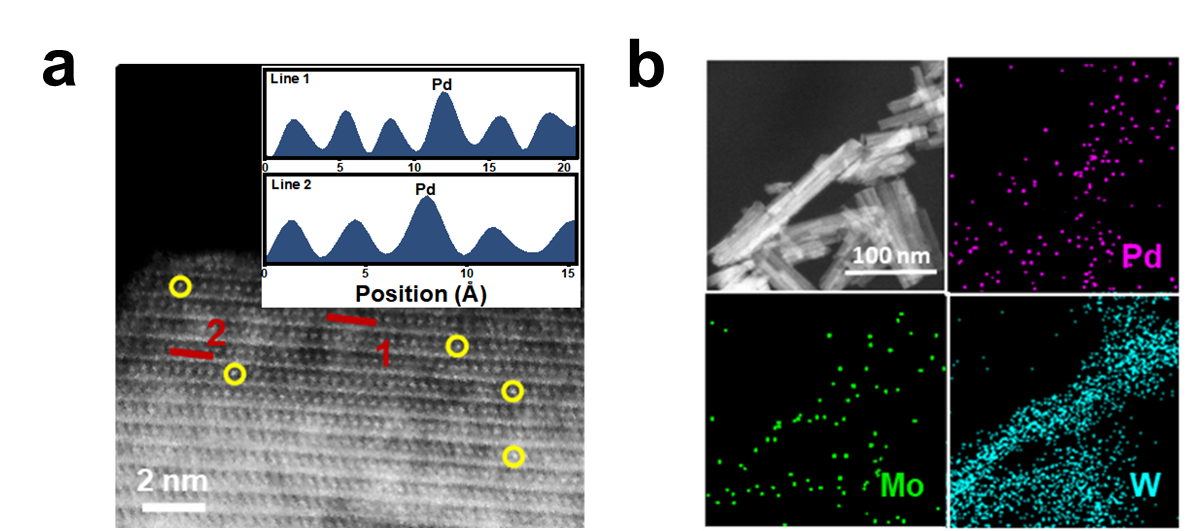


## Supplementary Figure 41. (a) Aberration-corrected HAADF-STEM image of Pd_1_/MoWO_3_, with single-site Pd marked by yellow circles. (b) EDX mapping images of different elements of Pd_1_/MoWO_3_.


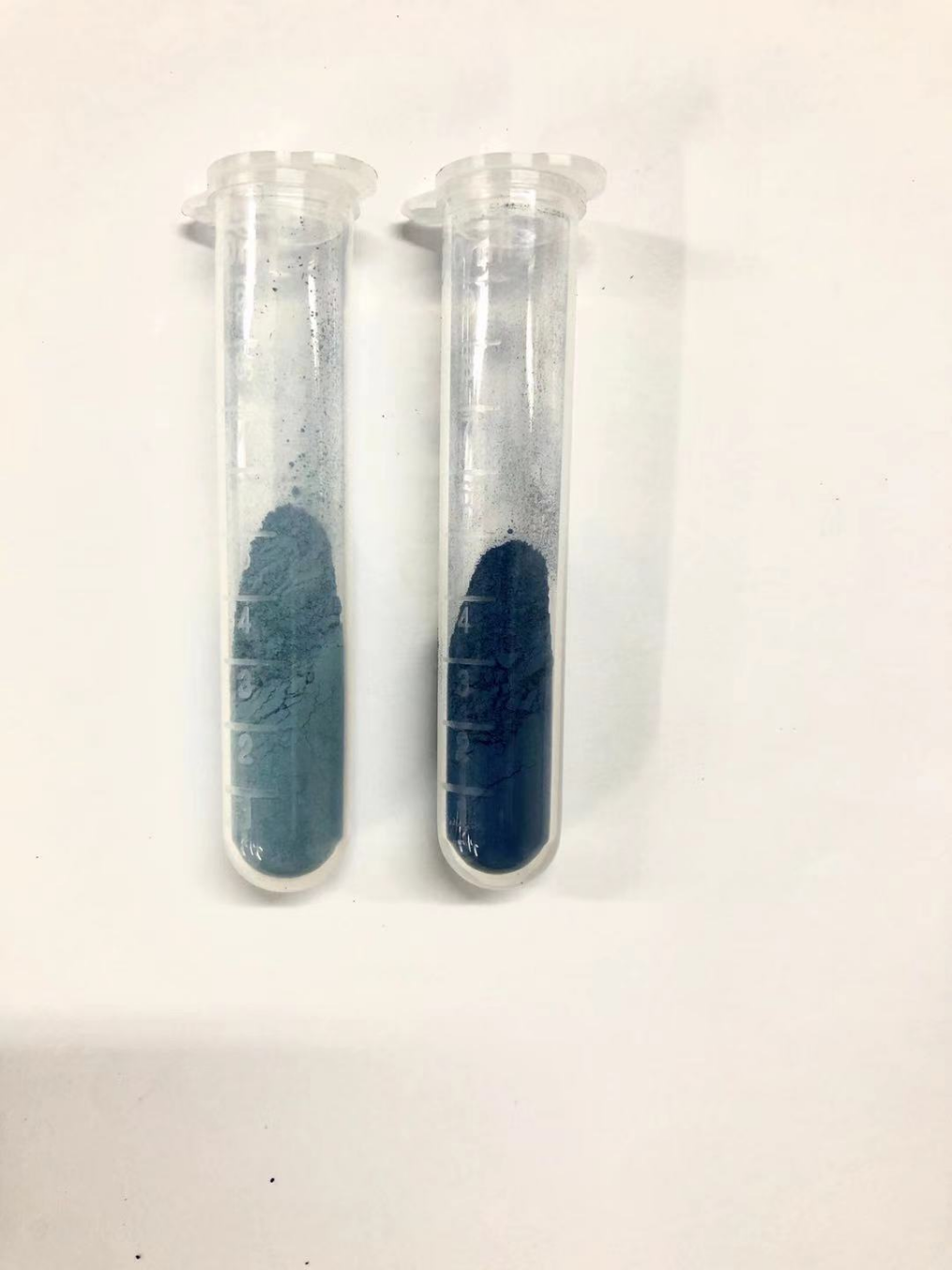


## Supplementary Figure 42. Photos of as-synthesized WO_3_ (the light blue powder in the left tube) and MoWO_3_ (the dark blue powder in the right tube).

## Supplementary Figure 43. EPR spectra of WO_3_ and MoWO_3_.

## Supplementary Figure 44. XPS spectra of Pd 3*d* for Pd_1_/WO_3_ and Pd_1_/MoWO_3_.


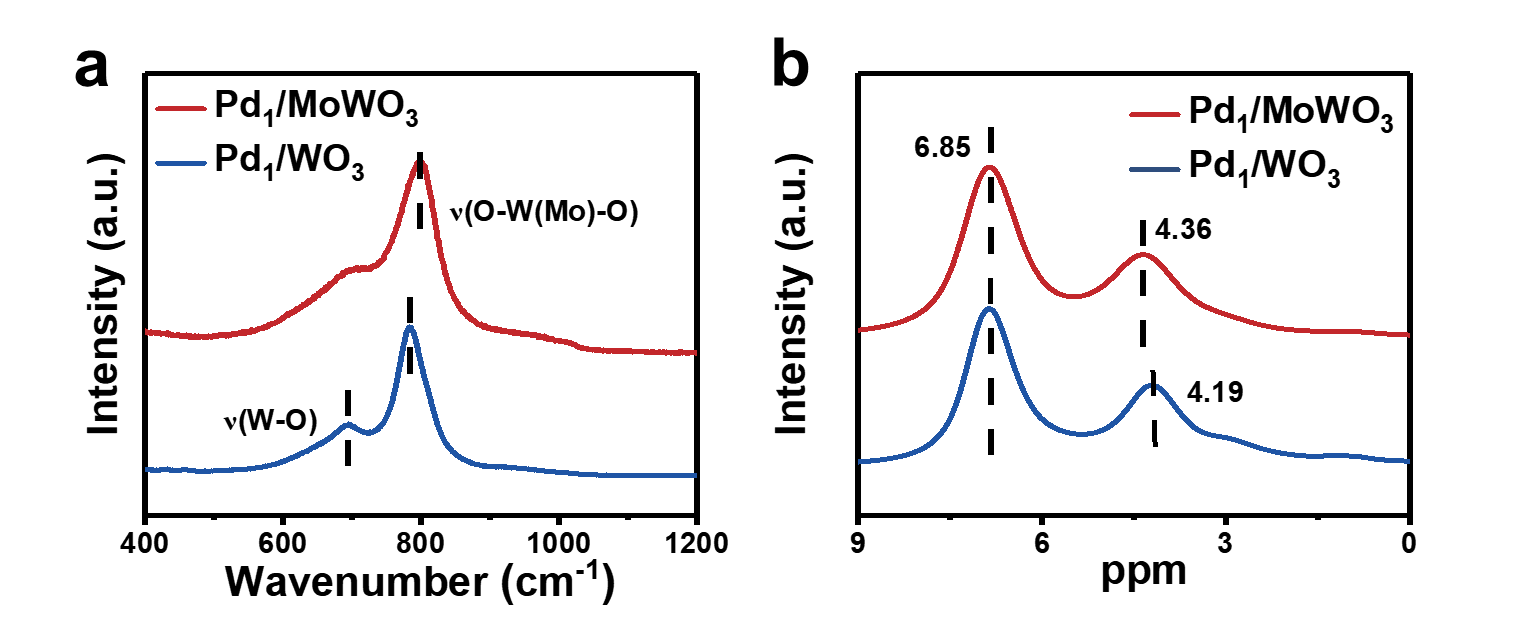


## Supplementary Figure 45. Raman spectra (a) and ^1^H solid-state NMR (b) of Pd_1_/WO_3_ and Pd_1_/MoWO_3_. As for Raman spectra, the red shift of the peak for O-W(Mo)-O vibration from 784 cm^-1^ to 797 cm^-1^ after Mo doping illustrated that the O-W-O bond in Pd_1_/MoWO_3_ was enhanced, which rendered the surface hydroxyl groups more prone to react. ^1^H solid-state NMR also confirmed the enhanced proticity of the hydrogen species as the positive shift of bridge hydroxyl groups


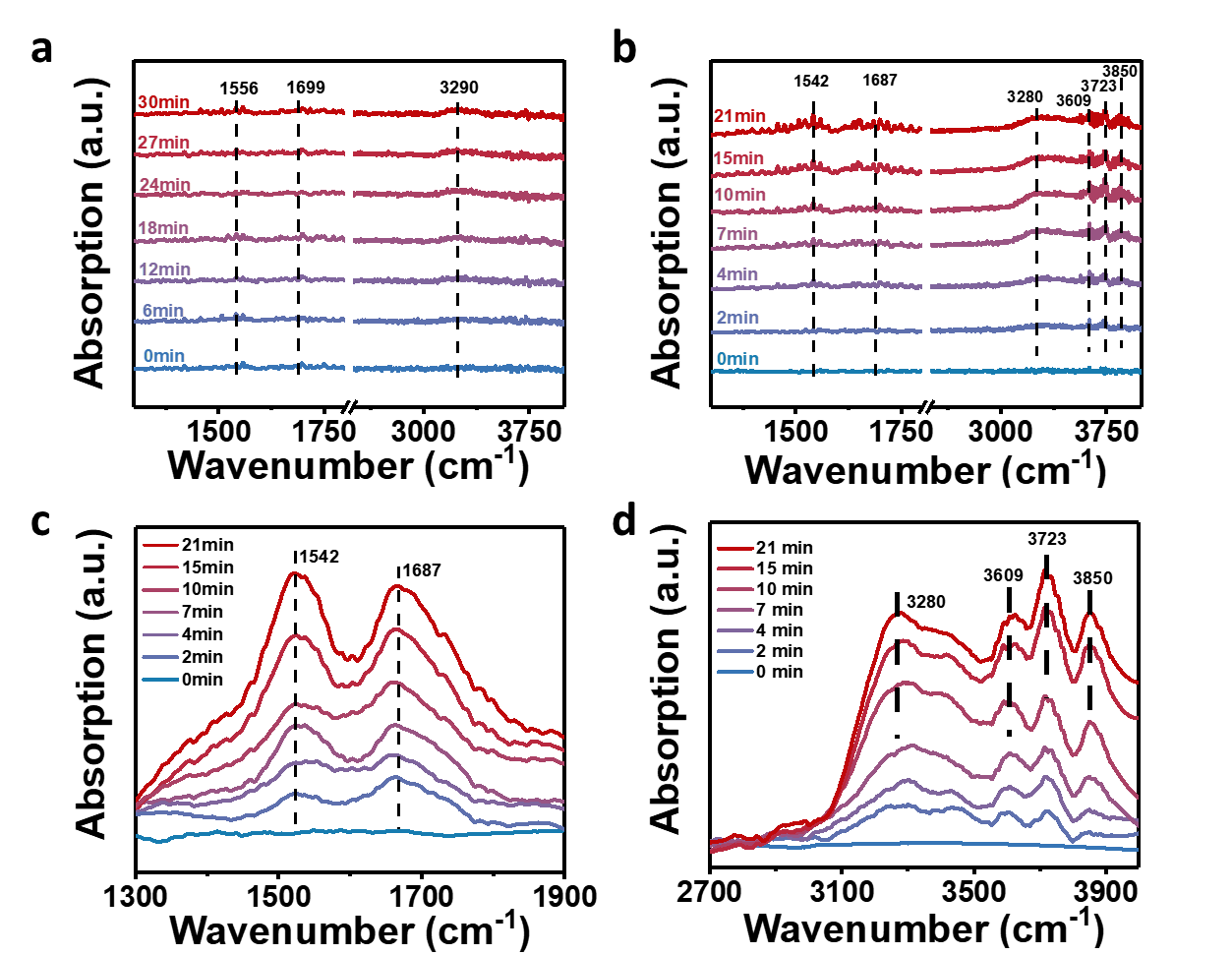


## Supplementary Figure 46. Evolution of in situ FT-IR spectra of H_2_ adsorbed on (a) Pd_1_/WO_3_ and (b) Pd_1_/MoWO_3_. (c, d) Magnified figure of the specific peaks at 1542 cm^-1^ and 3280 cm ^-1^ in the in situ FT-IR spectra of Pd_1_/MoWO_3_.

## Supplementary Figure 47. DFT calculations. Configurations and the calculated E_ads_ values of MBY and MBE adsorption on Pd_1_/MoWO_3_ and Pd_1_/MoWO_3_-H. Color code – dark blue: Pd, light blue: W, grey: C, red: O, white: H, cyan: Mo.


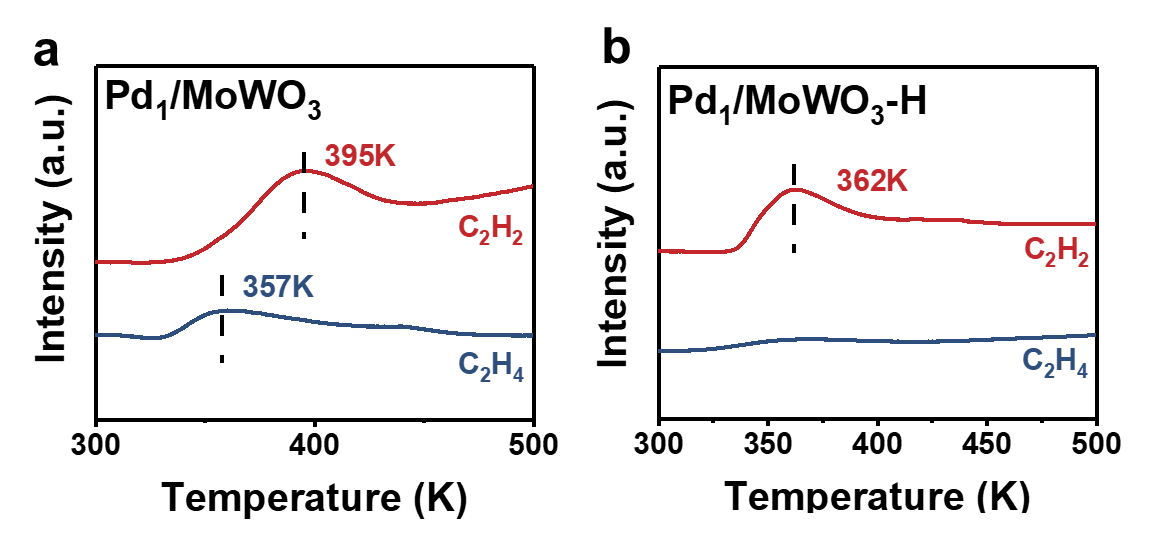


## Supplementary Figure 48. Temperature-programmed desorption experiments of C_2_H_2_ and C_2_H_4_ over (a) Pd_1_/MoWO_3_ and (b) Pd_1_/MoWO_3_-H.

## Supplementary Figure 49. Catalytic activity and selectivity for the selective hydrogenation of MBY over Pd_1_/WO_3_ and Pd_1_/MoWO_3_. Reaction condition: 10 ml of ethanol, 40 ℃, 1 bar H_2_, and 1000 rpm. 1 mmol substrate, 10 mg catalyst.


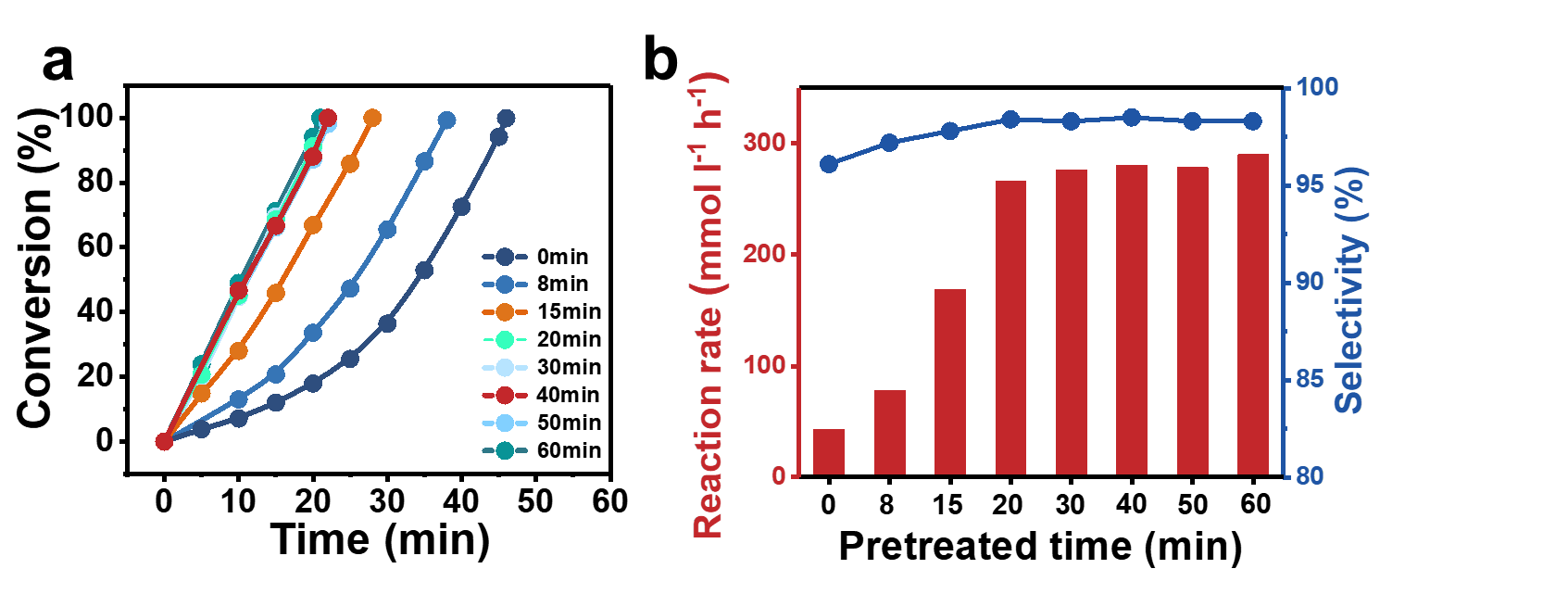


## Supplementary Figure 50. (a) The catalytic performance over Pd_1_/MoWO_3_ after pre-activation in H_2_ for different period. (b) Corresponding reaction rate and selectivity of semi-hydrogenation over Pd_1_/MoWO_3_ for different pretreated time.


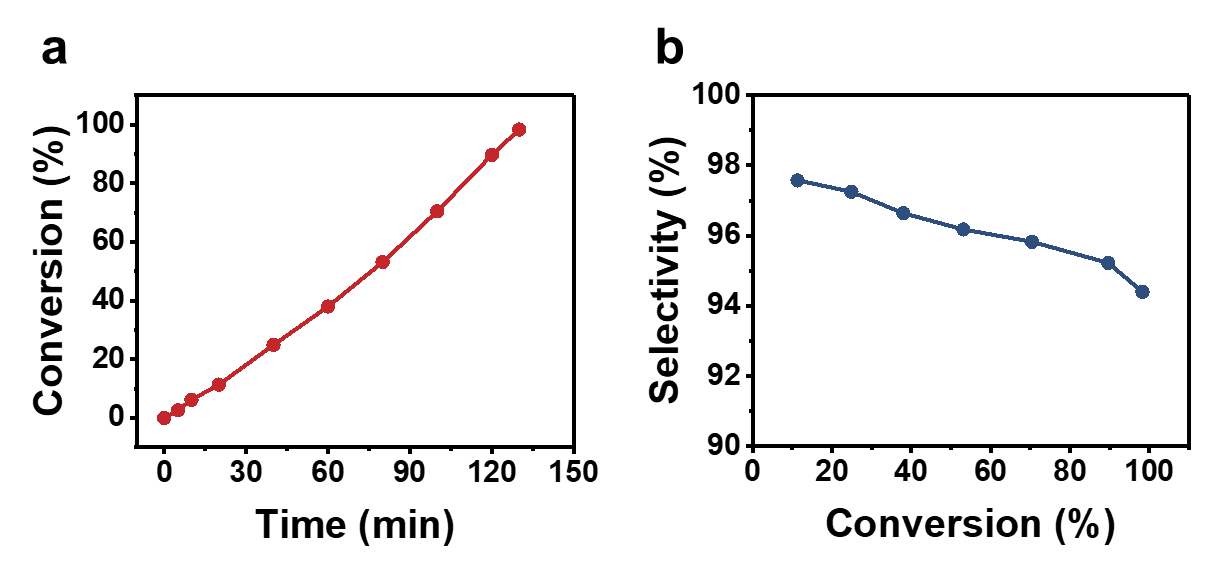


## Supplementary Figure 51. Catalytic activity (a) and selectivity (b) for the selective hydrogenation of MBY over commercial Lindlar catalyst.

***
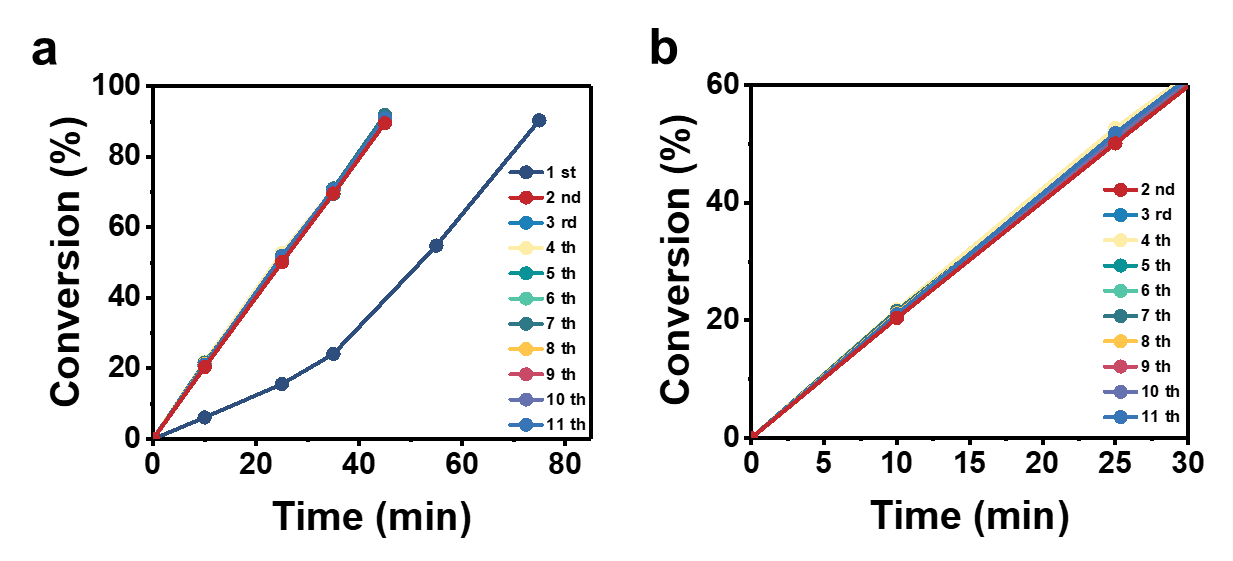
***

## Supplementary Figure 52. (a) Comparison of each cycle in terms of conversion versu time curves on Pd_1_/MoWO_3_. (b) Partial enlargement of the previous conversion versu time curve form 0 min to 30 min. Reaction condition: 10 ml of ethanol, 40 ℃, 1 bar H_2_, and 1000 rpm. 4 mmol substrate, 1.2*10^-3^ wt% Pd.


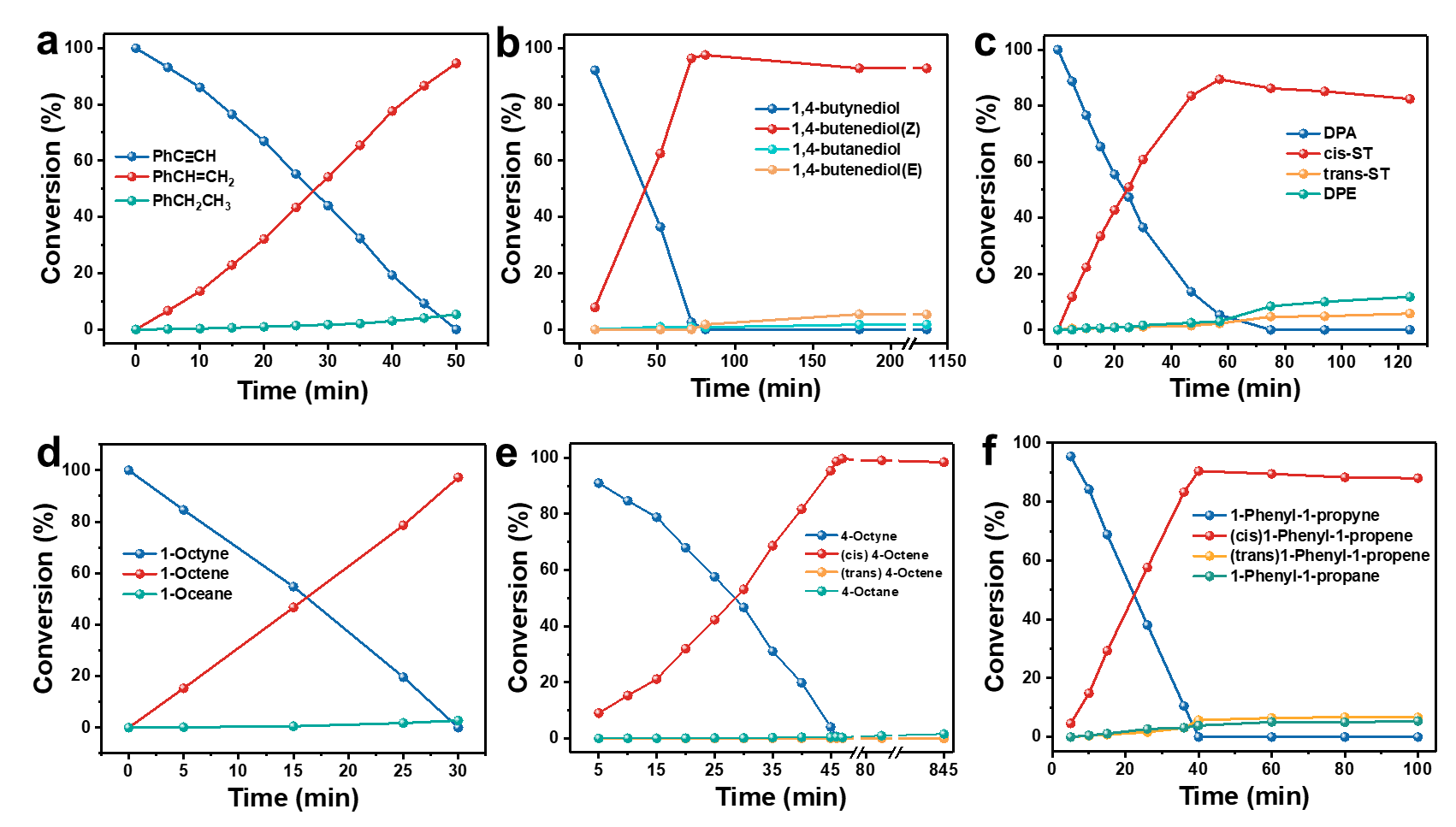


## Supplementary Figure 53. Catalytic activity of the hydrogenation of (a) phenylacetylene, (b) 1,4-butynediol, (c) diphenylacetylene, (d) 1-Octyne, (e) 4-Octyne, (f) 1-phenyl-1-prpyne over Pd_1_/MoWO_3_. Reaction condition: 10 mL of ethanol, 40℃, 1000 rpm, 1 mmol substrate, 0.3*10^-3^ wt% Pd.

## Supplementary Figure 54. Catalytic activity and selectivity for the selective hydrogenation of MBY over fresh and activated Pd_1_/MoWO_3_ without solvents. Reaction condition: 5 mL MBY, 1.5×10^-3^ wt % Pd, 80 ºC, 1 bar H_2_, 1000 rpm.

***
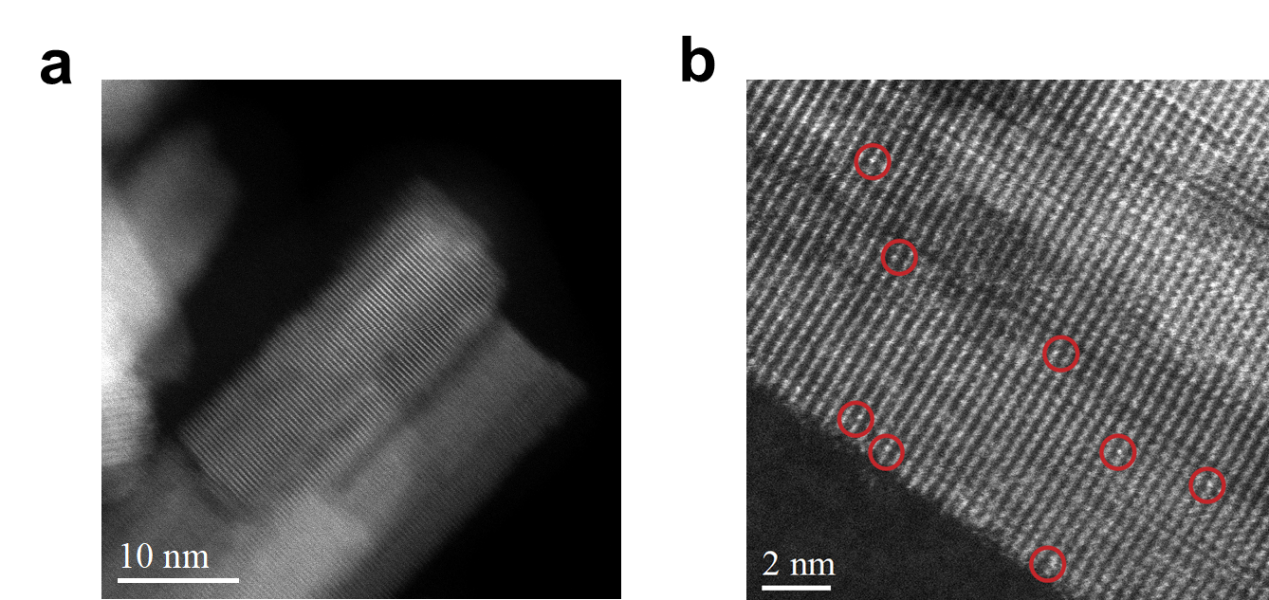
***

## Supplementary Figure 55. (a) Aberration-corrected HAADF-STEM image of Pt_1_/MoWO_3_. (b) Partial enlargement of the previous image with single-site Pt marked by red circles.

**
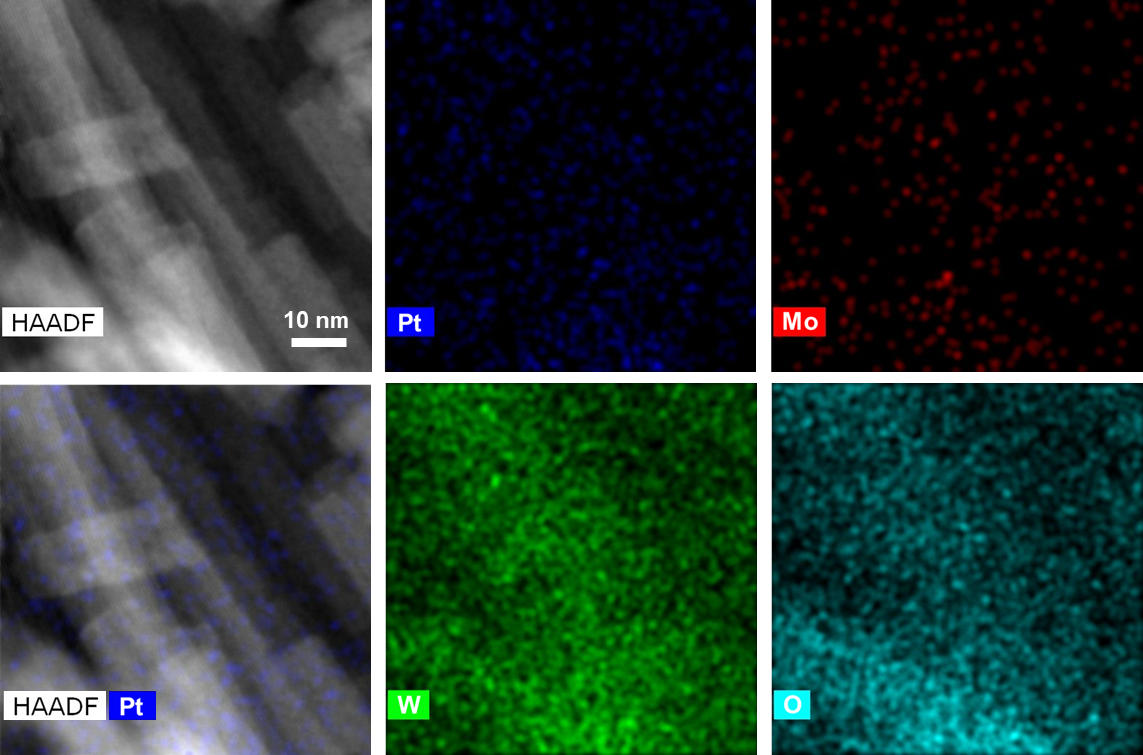
**

## Supplementary Figure 56. EDX mapping images of different elements of Pt_1_/MoWO_3_.

******

## Supplementary Figure 57. XRD pattern of Pt_1_/MoWO_3_.

# Supplementary Tables

| Sample | Shell | N | R(Å) | σ^2^×10^3^(Å^2^) | ΔE_0_(eV) | R-factor |
| --- | --- | --- | --- | --- | --- | --- |
| Pd foil | Pd-Pd | 12 | 2.74±0.01 | 5.30±0.01 | 3.90±0.03 | 0.007 |
| PdO | Pd-O | 5.5±0.6 | 2.01±0.01 | 4.21±0.56 | 1.64±2.01 | 0.011 |
|  | Pd-Pd | 11.2±3.0 | 3.00±0.02 | 8.92±0.22 | 1.72±2.54 |  |
|  | Pd-Pd | 6.8±2.5 | 3.45±0.01 | 4.67±0.89 |  |  |
| Pd_1_/WO_3_ | Pd-O | 1.9±0.3 | 2.00±0.01 | 2.64±0.01 | 1.32±2.19 | 0.014 |

## Supplementary Table 1. EXAFS fitting parameters at the Pd K-edge for samples. (*Ѕ*_0_^2^=0.80)

*S*_0_^2^ is the amplitude reduction factor; CN is the coordination number; R is interatomic distance (the bond length between central atoms and surrounding coordination atoms); σ^2^ is Debye-Waller factor (a measure of thermal and static disorder in absorber-scatterer distances); ΔE_0_ is edge-energy shift (the difference between the zero kinetic energy value of the sample and that of the theoretical model); R factor is used to value the goodness of the fitting.

## Supplementary Table 2. Catalytic performance of different catalysts for selective hydrogenation of MBY.

| Entry | Catalyst | Reaction condition | Conv. (%) | Sel. (%) | Ref |
| --- | --- | --- | --- | --- | --- |
| **1** | **Pd_1_/MoWO_3_-H** | **40 °C, 0.1 MPa, 0.1 M** | **99** | **98.4** | **This work** |
| **2** | **Pd_1_/MoWO_3_** | **40 °C, 0.1 MPa, 0.1 M** | **99** | **96.1** | **This work** |
| **3** | **Pd_1_/WO_3_-H** | **40 °C, 0.1 MPa, 0.1 M** | **99** | **95.4** | **This work** |
| **4** | **Pd_1_/WO_3_** | **40 °C, 0.1 MPa, 0.1 M** | **99** | **88.9** | **This work** |
| 5 | C-Pd/TiS | 30 °C, 0.3 MPa, 0.2 M | 97 | 95 | 2 |
| 6 | PdZn-ZnO/NCHS | 45 °C, 0.1 MPa, 0.14 M | 99 | 96 | 3 |
| 7 | PdBi/SiO_2_ | 50 °C, 0.1 MPa, 0.2 M | 100 | 94-96 | 4 |
| 8 | PdZn/TiO_2_ | 40 °C, 0.1 MPa, 0.1 M | 99 | 96.9 | 5 |
| 9 | PdIn/In_2_O_3_ | 30 °C, 0.1 MPa, 0.25 M | 99 | 95 | 6 |
| 10 | Pd/NHPC-DETA | 35 °C, 0.1 MPa, 0.25 M | 99 | 95 | 7 |
| 11 | PdZn/CN@ZnO | 35 °C, 0.5 MPa, 0.2 M | 99 | 95 | 8 |
| 12 | Ni_2_P | 30 °C, 0.5 MPa, 0.1 M | 99 | 92 | 9 |
| 13 | Pd/ZnO/SMF | 35 °C, 0.5 MPa, 0.1 M | 99 | 94.5 | 10 |
| 14 | Pd-PVP | 60 °C, 0.4 MPa, 0.34 M | 99 | 97 | 11 |
| 15 | PdZn-PVPone | 60 °C, 0.5 MPa, 0.1 M | 99 | 92.3 | 12 |

## Supplementary Table 3. Catalytic performance of Pd_1_/MoWO_3_ for a series of alkynes

| Entry*^a^* | Substrate | Structure | Reaction rate (mmol l^-1^ h^-1^) | Conv. (%) | Sel. (%) | Z/E |
| --- | --- | --- | --- | --- | --- | --- |
| 1 | Phenylacetylene |  | 81.768 | >99 | 95.1 |  |
| 2 | 1-Octyne |  | 183.6 | >99 | 99.2 |  |
| 3 | Benzyl propargyl ether |  | 90.25 | >99 | 96.2 |  |
| 4 | 4-ethynyltoluene |  | 176.21 | >99 | 95.8 |  |
| 5 | 1-ethynyl-4-methoxybenzene |  | 65.12 | >99 | 95.2 |  |
| 6 | 1-ethynyl-4-fluorobenzene |  | 152.21 | >99 | 96.4 |  |
| 7 | 4-Octyne |  | 108.2 | >99 | 99.6 | 99:1 |
| 8 | 1-Phenyl-1-propyne |  | 89.4 | >99 | 96.2 | 94:6 |
| 9 | Diphenylacetylene |  | 141.48 | >99 | 96.8 | 98:2 |
| 10*^b^* | 3-Hexyn-1-ol |  | 112.4 | >99 | 97.3 | 99:1 |
| 11*^c^* | 1,4-Butynediol |  | 48.6 | >99 | 99.2 | 99:1 |
| 12 *^c^* |  |  | 54.2 | >99 | 94.8 |  |
| 13 *^c^* |  |  | 23.1 | >99 | 92.3 |  |
| 14*^c^* |  |  | 43.2 | >99 | 92.8 | 93:7 |

*^a^*Reaction condition: 10 mL of ethanol, 40℃, 1 bar H_2_, and 1000 rpm. 1 mmol substrate, 0.028 mol % Pd. *^b^*Reaction condition: 10 mL of ethanol, 60℃, 1 bar H_2_, and 1000 rpm. 1 mmol substrate, 0.028 mol % Pd. *^c^*Reaction condition: 10 mL of ethanol, 80℃, 1 bar H_2_, and 1000 rpm. 1 mmol substrate, 0.084 mol % Pd.

## Supplementary Table 4. Selective hydrogenation of nitrobenzene with different substitutions.

| Catalyst | Reactant | Product | Reaction rate (mmol l^-1^ h^-1^) | Conv. (%) | Sel. (%) |
| --- | --- | --- | --- | --- | --- |
| Pd_1_/MoWO_3_ |  |  | 1.7 | >99 | 95.8 |
| Pd_1_/MoWO_3_-H |  |  | 2.8 | >99 | 98.9 |
| Pt_1_/MoWO_3_ |  |  | 8.9 | 98.9 | 64.9 |
| Pt_1_/MoWO_3_-H |  |  | 12.4 | 99.1 | 83.2 |

Reaction condition: 10 ml of ethanol, 40 °C, 1 Mpa H_2_, and 1000 rpm. 1 mmol substrate, 0.056 mol% Pd (Pt).

## Supplementary Table 5. The loadings of Pd of different catalysts with the average Pd size of 2.8 nm estimated using an ICP spectrometer

| Entry | Catalyst | Theoreticl Pd loading（wt%） | Pd weight loading（wt%） |
| --- | --- | --- | --- |
| 1 | Pd/Al_2_O_3_ | 0.30 | 0.29 |
| 2 | Pd/SiO_2_ | 0.30 | 0.30 |
| 3 | Pd/MgO | 0.30 | 0.28 |
| 4 | PdTiO_2_ | 0.30 | 0.29 |
| 5 | Pd/CeO_2_ | 0.30 | 0.30 |
| 6 | Pd/WO_3_ | 0.30 | 0.30 |

| Entry | Catalyst | Theoreticl Pd loading（wt%） | Pd weight loading（wt%） |
| --- | --- | --- | --- |
| 1 | Pd/Al_2_O_3_ | 0.30 | 0.29 |
| 2 | Pd/SiO_2_ | 0.30 | 0.30 |
| 3 | Pd/MgO | 0.30 | 0.28 |
| 4 | PdTiO_2_ | 0.30 | 0.29 |
| 5 | Pd/CeO_2_ | 0.30 | 0.30 |
| 6 | Pd/WO_3_ | 0.30 | 0.30 |

| Catalyst | Particle size (nm) | Theoreticl Pd loading（wt%） | Pd weight loading（wt%） |
| --- | --- | --- | --- |
| Pd/WO_3_ | Single atom | 0.05 | 0.046 |
|  |  | 0.10 | 0.096 |
|  |  | 0.20 | 0.192 |
|  |  | 0.30 | 0.295 |
|  | 2.8 | 0.05 | 0.053 |
|  |  | 0.10 | 0.104 |
|  |  | 0.20 | 0.206 |
|  |  | 0.30 | 0.303 |
|  | 4.6 | 0.05 | 0.049 |
|  |  | 0.10 | 0.095 |
|  |  | 0.20 | 0.197 |
|  |  | 0.30 | 0.289 |
|  | 7.8 | 0.05 | 0.054 |
|  |  | 0.10 | 0.106 |
|  |  | 0.20 | 0.207 |
|  |  | 0.30 | 0.308 |

## Supplementary Table 6. The loadings of Pd of single atom catalysts and catalyst with the average Pd size of 2.8 nm, 4.6 nm and 7.8 nm estimated using an ICP spectrometer.

# Supplementary References

1 Murata, K. et al*.* Exploiting metal–support interactions to tune the redox properties of supported Pd catalysts for methane combustion. *ACS Catal.* **10**, 1381-1387 (2020).

2. Witte, P. T. et al. BASF NanoSelect™ technology: innovative supported Pd- and Pt-based catalysts for selective hydrogenation reactions. *Top. Catal.* **55**, 505-511 2012.

3. Ye, C. et al. PdZn intermetallic compound stabilized on ZnO/nitrogen-decorated carbon hollow spheres for catalytic semihydrogenation of alkynols. *Nano Res.* **15**, 3090-3098 (2022).

4. Cherkasov, N. et al. Palladium–bismuth intermetallic and surface-poisoned catalysts for the semi-hydrogenation of 2-methyl-3-butyn-2-ol. *Appl. Catal. A Gen.* **497**, 22-30 (2015).

5. Okhlopkova, L., Prosvirin, I., Kerzhentsev, M. & Ismagilov, Z., Selective Hydrogenation of 2-Methyl-3-butyn-2-ol in Microcapillary Reactor on Supported Intermetallic PdZn Catalyst, Effect of Support Doping on Stability and Kinetic Parameters. *Catalysts* **12**, 1660 (2022).

6. Mao, S. et al. Tuning the catalytic performance for the semi-hydrogenation of alkynols by selectively poisoning the active sites of Pd catalysts. *Green Chem.* **21**, 4143-4151 (2019).

7. Shen, L. et al. PdZn intermetallic on a CN@ZnO hybrid as an efficient catalyst for the semihydrogenation of alkynols. *J. Catal.* **350**, 13-20 (2017).

8. Luo, Q. et al. Huang, G.; Li, H.; Wang, Y., Dynamic Modification of Palladium Catalysts with Chain Alkylamines for the Selective Hydrogenation of Alkynes. *ACS Appl. Mater. Interfaces* **13**, 31775-31784 (2021).

9. Albani, D. et al. Ensemble Design in Nickel Phosphide Catalysts for Alkyne Semi‐Hydrogenation. *ChemCatChem* **11**, 457 (2019).

10. Semagina, N., Grasemann, M., Xanthopoulos, N., Renken, A. & Kiwi-Minsker, L., Structured catalyst of Pd/ZnO on sintered metal fibers for 2-methyl-3-butyn-2-ol selective hydrogenation. *J. Catal.* **251**, 213-222 (2007).

11. Artur et al. Size-effect of Pd-(poly(N-vinyl-2-pyrrolidone)) nanocatalysts on selective hydrogenation of alkynols with different alkyl chains. *J. Phys. Chem. C* **117**, 13424-13434 (2013).

12. Okhlopkova, L., Matus, E. V., Prosvirin, I. P., Kerzhentsev, M. A. & Ismagilov, Z. R. Selective hydrogenation of 2-methyl-3-butyn-2-ol catalyzed by embedded polymer-protected PdZn nanoparticles. *J Nanopart Res* **17**, 475 (2015)..
